# Acute effects of a single dose of 2 mA of anodal transcranial direct current stimulation over the left dorsolateral prefrontal cortex on executive functions in patients with schizophrenia—A randomized controlled trial

**DOI:** 10.1371/journal.pone.0254695

**Published:** 2021-07-16

**Authors:** Thomas M. Schilling, Magdalena Bossert, Miriam König, Gustav Wirtz, Matthias Weisbrod, Steffen Aschenbrenner

**Affiliations:** 1 Section of Clinical Psychology and Neuropsychology, SRH Clinic Karlsbad-Langensteinbach, Karlsbad, Germany; 2 SRH Psychiatric Rehabilitation Center, Karlsbad, Germany; 3 Department of Psychiatry and Psychotherapy, SRH Clinic Karlsbad-Langensteinbach, Karlsbad, Germany; 4 Department of General Psychiatry, Center of Psychosocial Medicine, University of Heidelberg, Heidelberg, Germany; Ruhr University Bochum International Graduate School of Neuroscience, GERMANY

## Abstract

**Objective:**

Cognitive impairments are a frequent and difficult to treat symptom in patients with schizophrenia and the strongest predictor for a successful reintegration in occupational and everyday life. Recent research suggests transcranial direct current stimulation (tDCS) to enhance cognition in this patient group. However, the question regarding its acute effectiveness on executive functions remains largely unanswered. Here, we examined in a randomized, double blind, sham-controlled repeated-measures design the impact of tDCS on performance in several executive functions in patients with schizophrenia, schizoaffective disorder or acute transient psychotic disorder.

**Methods:**

Patients (N = 48) were tested twice using standardized, well-constructed and clinically validated neuropsychological tests assessing verbal working memory, response inhibition, mental flexibility and problem solving. In session 1 they solely underwent the neuropsychological assessment, whereas in session 2 they additionally received 2 mA of anodal tDCS stimulation over the left dorsolateral prefrontal cortex (DLPFC), cathode right supraorbital ridge, or sham stimulation for 20 minutes.

**Results:**

Patients of both groups were not able to correctly discriminate the type of stimulation received confirming the success of the blinding procedure. However, analyzing the whole sample the change in performance from session 1 to session 2 was the same in the verum as in the sham condition (all *p* >.5). Moreover, a subsequent exploratory analysis showed that performance in the response inhibition task was worse for patients that engaged in the task within 20 minutes after the end of the verum stimulation.

**Conclusion:**

Hence, 2 mA of anodal tDCS applied over the left DLPFC did not acutely enhance executive functions in patients with schizophrenia or related disorders but impaired performance in the response inhibition task shortly after. Future studies should continue to seek for effective stimulation configurations for this patient group.

**Clinical trial registration:**

The study is registered in the “Deutsches Register Klinischer Studien DRKS”, German Clinical Trial Register and has been allocated the following number: DRKS00022126.

## 1. Introduction

Schizophrenia is a heterogeneous, debilitating neuropsychiatric disorder affecting about 1% of the population worldwide. Aside to its core symptoms such as delusions, hallucinations, paranoia and aberrations in speech and behavior cognitive impairments are a hallmark of the disease [1]. Importantly, while acute psychotic symptoms often improve under proper antipsychotic medication, cognitive dysfunctions frequently persist independent of acute psychotic phases and up to now remain most difficult to treat [2, 3]. In line with this, cognitive impairments are the strongest predictor for a successful reintegration of a patient in everyday and occupational life [4]. Thus, the development of new treatment strategies targeting cognitive dysfunctions in patients suffering from schizophrenia is of utmost importance.

Patients with schizophrenia often display a variety of cognitive impairments in several neurocognitive domains such as attention, memory and executive functions [5, 6]. Executive functions are subdivided into several sub functions. In a prominent model Miyake and colleagues [7] suggest that executive functions consist of three basic mechanisms: (I) shifting (reallocation of the focus of attention), (II) updating (especially of working memory content), and (III) inhibition (of irrelevant but highly automatic response propensities). These basic mechanisms are considered to form the basis of even more complex executive functions such as problem solving or decision-making [8, 9]. Dysfunctions in the executive domain in patients with schizophrenia comprise disturbances in several basic (e.g., working memory, [10]) as well as more complex executive functions (e.g. problem solving, goal maintenance, rule generation and selection, dynamic adjustments and control, [6, 11–13]).

Executive functions have been linked to the structural and functional integrity of the prefrontal cortex (e.g. [14, 15]) and aberrations in both structure [16] and functioning in this brain region have frequently been reported in patients with schizophrenia. Particularly, it was demonstrated that compared to healthy controls patients with schizophrenia exhibit reduced neuronal activation at rest [17] as well as inefficient neuronal recruitment during working memory tasks in the dorsolateral prefrontal cortex (DLPFC) [18]. Given this association between neurophysiological alterations and cognitive impairments, it seems possible that a restoration of proper neurophysiological functioning of the DLPFC might enhance cognitive functions in this patient group. A promising method to modulate and improve neuronal and thereby cognitive functioning is transcranial direct current stimulation (tDCS).

Transcranial direct current stimulation is a safe [19] and comparatively easily applied neurostimulation technique, which is increasingly used in the treatment of numerous neurological and psychiatric disorders [20]. Briefly, it works by a direct current flow from a positively charged anodal to a negatively charged cathodal surface electrode mounted on the head or an extracephalic side. It´s acute effects rely on a membrane potential shift beneath the surface electrodes induced by the direct current flow resulting in a depolarization below the anodal and a hyperpolarization below the cathodal electrode [21, 22]. Importantly, tDCS itself does not induce action potentials but acts as a subthreshold technique thereby modulating cortical network excitability [23]. Modulations of excitability at the primary motor cortex last for at least 30 Minutes following stimulation depending on stimulation intensity, duration, and polarity [24]. The therapeutic effect of tDCS is suggested to rely on brain excitability modulation and/or inducing neuroplasticity. This indeed, could possibly enhance the ability of a neuronal network, for example the prefrontal cortex, to process incoming stimuli more efficiently during active task engagement.

The evidence of tDCS to acutely restore neurophysiological and thereby neurocognitive and specifically executive function in patients with schizophrenia is still limited. So far there is evidence for an acute enhancement of verbal working memory performance during [25] or shortly after [26] tDCS stimulation over the left DLPFC, albeit discrepant results have also been reported [27, 28]. Moreover, there is some evidence for an improvement of mental flexibility during stimulation of the left DLPFC [28]. Indeed, demonstrating that tDCS could also improve inhibition and shifting (i.e. mental flexibility) and conclusively problem solving and decision making would broaden its therapeutic range and thereby its clinical use for this patient group.

We designed the current experiment to extend our understanding of tDCS effects on executive functions in patients with schizophrenia. Following the taxonomy of Miyake [7] we selected one neuropsychological test for each sub executive function as well as one for one higher-order function, and assessed verbal working memory (updating), response inhibition (inhibition), mental flexibility (shifting) and problem solving. Patients were tested twice according to a randomized, double blind, sham-controlled repeated-measures design and received either 2mA of anodal tDCS or sham over the left DLPFC (cathode right supraorbital ridge) for 20 minutes in the second session. We chose to stimulate the left DLPFC based on the above stated evidence for functional impairment and promising tDCS effects in this region in patients with schizophrenia as well as on broader neuroanatomical considerations: the (left) DLPFC has frequently been associated with (verbal) working memory [29], decision making [14], relational integration and strategy switching [30] and also to a lesser extent with self-control and response inhibition [31].

Our main hypothesis was that patients receiving the verum stimulation would outperform patient receiving the sham stimulation in session II.

## 2. Material and methods

### 2.1 Trial design, randomization procedure and ethical approval

The trial was as double blind, randomized, controlled, prospective study. After inclusion in the study, participants were randomly assigned to one of two groups (A or B) following a randomization sequence. The only constrain in the randomization sequence was that within blocks of four positions in the sequence each condition (A or B) would occur two times. The performing experimenters who allocated participants to the groups, applied the tDCS-stimulation or performed the neuropsychological testing knew whether participants were in condition A or B, but were not aware whether group A or group B coded for verum or sham stimulation. Before each tDCS-application the experimenter entered a code which was provided by the manufacturer of the tDCS-device and that programmed either an A or B stimulation. Blinding of the experimenters was kept up until all data was collected and only then it was revealed whether A or B had coded for verum or sham stimulation during the study. Participants were also not aware of the kind of stimulation (verum or sham) they received. Thus, both experimenters and participant were fully blinded until the end of the study. The study and its procedures were approved by the Ethics Committee of the Faculty of Medicine of the University of Heidelberg, Germany (approval number: S-087/2018) and were in line with the latest revision of the Declaration of Helsinki. The study is registered in the “Deutsches Register Klinischer Studien DRKS”, German Clinical Trial Register and has been allocated the following number: DRKS00022126.

### 2.2 Sample and inclusion and exclusion criteria

The clinical sample was recruited from the Department of Psychiatry and Psychotherapy at the SRH Clinic Karlsbad-Langensteinbach, Germany as well as from neighboring educational and rehabilitation institutions (SRH Psychiatric Rehabilitation Center, Karlsbad, Germany; SRH Occupational Rehabilitation Center, Karlsbad, Germany). Patients (age range = 18–65) with an ICD-10 diagnosis of schizophrenia (ICD-10: F20), schizoaffective disorder (ICD-10: F25) or acute transient psychotic disorder (ICD-10: F23) were informed about the study and its procedures, asked whether they wanted to participate and if interested and eligible to give written informed consent. Experienced and board-certified psychiatrist or clinical psychologists confirmed the diagnoses of all patients according to ICD-10 criteria. Patients were excluded if they were minors, were of legal age but were unable to give their informed consent, had an impaired intelligence (i.e. IQ < 85), had used drugs in the last 8 weeks, were suffering from any central nervous system disorder, had an history of skull or heart surgery, fragments of metal in the skull or skin irritations at the forehead. Patients were allowed to take their regular medication, including antipsychotic medication, at the time of inclusion in the study except for an acute intake of benzodiazepines.

### 2.3 Procedure

The study consisted of two sessions separated by an interval of about one week (median: 7 days). In the first session, participants underwent the routine clinical neuropsychological assessment and filled in some extra questionnaires (see below). The duration of the first session was about 90 minutes. During the second session tDCS electrodes were attached to participants’ heads. Then the stimulation was started together with the first neuropsychological test and ran 20 minutes before the device automatically terminated it. During stimulation, participants repeated four of the neuropsychological tests already performed in the first session, which altogether took about 40 minutes. The stimulation, thus, ran only about half the time of the neuropsychological testing. However, due to the counterbalancing of the sequence of tests in the whole sample (see below) each neuropsychological test in each condition (verum/sham) was equally often performed during (“online”) or shortly after (“offline”) stimulation. Given that tDCS’ effects of different intensities including 2 mA last for at least 30 minutes [24] we assumed the effects to still be in place even 20 minutes after the end of the stimulation. Following the completion of testing participants filled in a questionnaire concerning the stimulation, were thanked for participating and received a small compensation. The second session lasted about 60 minutes.

### 2.4 Neuropsychological assessment and questionnaires

During routine clinical neuropsychological assessment in the first session all participants underwent a standardized test-battery of neuropsychological tests [32] comprising different subtests assessing attention, executive functions and memory as well as one additionally test of mental flexibility [33]. During the stimulation in the second session participants repeated four of the executive functions tests assessing problem solving [34], verbal working memory [35], response inhibition [36] and mental flexibility [33]. The tests were selected according to the aforementioned model of Miyake and colleagues [7, 8].

The problem solving test is a digital version of the Tower of London [34]. Briefly, the test is a spatial planning and problem solving test: it consists of three rods of different heights that can hold a different number of balls, and three balls of different colors. For each problem two pictures are presented: one picture depicts the goal state whereas the other picture shows the start state. The participant is asked to change the configuration of the balls in the start picture to the configuration of the goal picture using the minimal number of moves necessary which is the main dependent variable. There is a time limit imposed of one minute per problem. The verbal working memory task [35] was a 2-back task. Participants were presented with a sequence of 100 letters with a presentation time of 1.5 seconds and an interstimulus interval of 1.5 seconds. The letters in the sequence are repeated from time to time and participants are instructed to press a key as fast as possible whenever the presented letter matches the one presented two positions before. In the response inhibition task [36] participants are instructed to react by button push to a black triangle shown on the screen and to withhold a reaction if a black circle is presented. The stimuli are presented for 200 ms with an interstimulus interval of 1 second. In total 101 triangles and 24 circles are presented in random order. The higher frequency of the triangles establishes a response tendency which needs to be inhibited if the less frequent circles are presented. The mental flexibility test [33] is based on an alternating run paradigm. Participants are asked to either react to the color (bright vs. dark) or the shape (triangle vs. circle) of a geometric figure by pressing a left or right button depending on the target shown and the current rule in place. The rule (i.d. reacting to either color or shape) changes every second trial. See S1 Fig for a graphical depiction of the four neuropsychological tests used.

While the sequence of the neuropsychological tests on the first day was standardized by the test-battery and the same for all participants it was counterbalanced on the second session to avoid effects of sequence. All neuropsychological tests were presented on a standard flat panel monitor of 22 inch (resolution 1680 x 1050) by the Vienna Test System (Schuhfried, Mödling, Austria) and participants responses were recorded with the Vienna Testing Systems Keyboard or via mouse. Premorbid verbal intelligence was estimated using a German word selection test [37]. Self-reported depression was assessed using the German version of Beck Depression Inventory II [38]. The questionnaire following the stimulation was self-designed and assessed participants´ guessing whether they received verum or sham as well as the confidence of this rating and ratings of pain during the stimulation (visual analog scale from 0 to 100).

### 2.5. Stimulation

Transcranial direct current stimulation was applied with the *DC-Stimulator Mobile* (NeuroConn, Ilmenau, Germany) in a double blind fashion using the built-in study protocol of the device. Electrodes (size: 5x5 cm/25 cm^2^) were inserted in sponge pads (size: 5x5 cm/25 cm^2^) soaked in saline solution (NaCl 0.9%) before they were attached to participants’ heads with the help of rubber strings. The anode electrode was positioned at F3, the cathode electrode at FP2 according to the international 10–20 EEG-system. In the verum condition participants received a stimulation of 2mA for 20 minutes (8s fade in, 1200s tDCS 2mA, 8s fade out; total duration 1216s). In the sham condition a true stimulation was simulated in the beginning before the device reduced the applied current after 40 seconds (8s fade in, 40s tDCS 2mA, 8s fade out, 1120s sinus 85 Hz 50 μA; total duration 1216s).

### 2.6 Data reduction, statistical analysis and sample size calculation

#### 2.6.1 Data reduction

After data screening for outliers and invalid data entry the raw values of each neuropsychological test were standardized (t-scored) to the whole sample jointly to session 1 and session 2. Each performance measure of each neuropsychological test (e.g. reaction time, correct responses) was standardized separately. Standardized data was checked for outliers, which were truncated to ±2 *SD* from the mean of the whole sample, and was used for further analysis. To assess performance in the working memory test aside from reaction time the signal detection variables *d-prime* and *response criteria C* were calculated using hits and false alarms as described elsewhere [39]. All statistical analyses were calculated using SPSS 25 (IBM SPSS statistics) with a critical *α*-level of *p* = 0.05 (two-tailed) per model.

#### 2.6.2 Main analysis

To screen for potential baseline differences in session 1 which could have potentially influenced stimulation reactivity in session 2 simple paired t-tests between both groups (“verum”, “sham”) were calculated for neuropsychological performance in session 1 for each performance measures of each neuropsychological test.

To assess the impact of stimulation on neuropsychological performance a separate 2 TIME (“session 1” vs. “session 2”)* GROUP (“verum”, “sham”) mixed-model repeated-measures ANOVA was calculated for each performance measures of each neuropsychological test. We expected to observe TIME*GROUP interactions indicating differential changes in neuropsychological performance from session 1 to session 2 as a function of kind of stimulation. Data of questionnaires was analyzed using non-parametric Man-Whitney-U-Tests (ratings concerning pain, ratings concerning confidence of guessing of received stimulation,) or χ2 tests (guessing of received stimulation).

We a priori calculated the sample size for the main analysis using the program GPower [40] for a power of 1 -*β* = 0.9. with a critical α-level of *p* = 0.05 (two-tailed), an assumed correlation among repeated measures of 0.5 for an expected clinically meaningful effect size of *f* = 0.25, equivalend to *d* = 0.5 [41] for the TIME*GROUP interaction to be 23 participants per group.

#### 2.6.3 Exploratory analysis

To assess whether performance in the neuropsychological tests differed in participants that received a specific test “online” (i.e. during stimulation) as compared to “offline” (i.e. after stimulation) we performed an additional exploratory analysis. A test which had been performed approximately within the first 20 minutes of the neuropsychological testing and therefore while current was applied was considered to be “online”, whereas a test which had been performed within the second 20 minutes of the neuropsychological testing was considered to be “offline”. For each dependent variable the sample was split into two groups (i.d. participants that had performed the specific neuropsychological test “online” vs. “offline”) with N = 24 in total per exploratory analyses and n = 12 per condition (sham vs. verum) and within each group separate 2 TIME (“session 1” vs. “session 2”)* GROUP (“verum”, “sham”) mixed-model repeated-measures ANOVAs were calculated for each dependent variable. As for the main analysis, we expected to observe a TIME*GROUP interaction and restrict the presentation of data of the exploratory analysis to the interaction effect.

## 3. Results

### 3.1 Enrollment and finally analyzed sample

**Fig 1:**
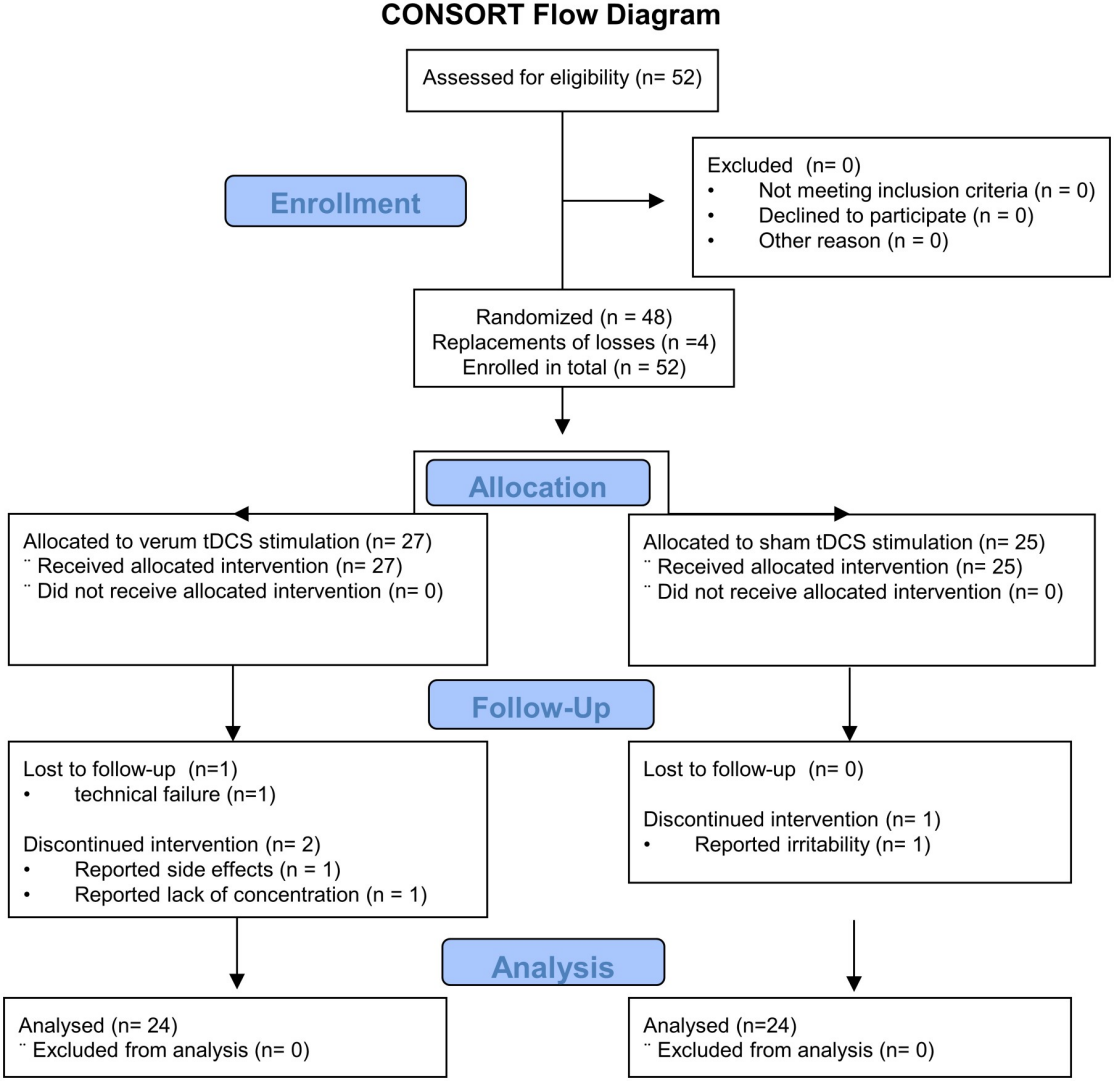
CONSORT flow diagram of the study. CONSORT Flow Diagram of the study giving an overview of the participants included in the study and finally analyzed.

In total 52 patients agreed and were eligible to participate and were included in the study. Three participants aborted the experiment in the second session shortly after the beginning of the stimulation, one due to reported adverse side effects of the stimulation (group verum = 1), one due to a reported lack of concentration (group verum = 1), one due to reported irritability (group sham = 1). Data of one further participant (group verum = 1) was lost due a technical failure of the CPU running the neuropsychological testing system. All four participants were replaced as to maintain the counterbalancing in the sequence of the neuropsychological tests and to achieve an equal sample size in both groups. Enrollment in the study took part between May 2018 and July 2019. The study was ended after both groups had reached the *a priori* estimated sample size and counterbalancing of the neuropsychological tests in both groups was achieved (which necessitated n = 24 per group.) The final sample size finally included in the study and the final analysis’s consisted of N = 48 participants (9 females). See Fig 1 for a CONSORT flow-chart of enrollment and finally analyzed sample in the study.

### 3.2 Demographic and clinical data

The majority of patients included in the final analysis fulfilled diagnostic criteria for schizophrenia (ICD-10: F20, N = 40), the other for schizoaffective disorder (ICD-10: F25, N = 7) and one for acute transient psychotic disorder (ICD-10: F23.1). 14 patients (7 in both groups) were diagnosed with one or more comorbid psychiatric disorders. Patients of both groups did not differ in age, sex, duration of disease, handedness, current intake of antipsychotic medication, smoking, self-reported depression or estimated premorbid verbal intelligence. See Table 1 for an overview.

**Table 1:** Demographic and clinical data.

	verum group		sham group	
**N total**	24		24	
**N females**	5		4	
**ICD-10: F20**	20/24		20/24	
**ICD-10: F23.1**	1/24		0/24	
**ICD-10: F25**	3/24		4/24	
**Age Mean**	*M* = 28	*SD* = 7	*M* = 31	*SD* = 9
**Duration of Disease (Month)**	*M* = 30.91	*SD* = 55.43	*M* = 42.89	*SD* = 96.15
**Antipsychotic medication**	23/24		22/24	
**Right handedness**	19/20		16/19	
**Smoking**	14/24		16/24	
**BDI-II (SD)**	*M* = 14	*SD* = 6	*M* = 18	*SD* = 14
**MWT (SD)**	*M* = 103	*SD* = 10	*M* = 105	*SD* = 16

Demographic data separated by group including number of participants, sex, diagnostic category, age, duration of disease (data available for 38/48 participants), status of antipsychotic medication, handedness (data available for 39/48 participants), smoking as well as self-reported depression (BDI-II) and estimation of premorbid verbal intelligence (MWT-B). For age, duration of disease, self-reported depression and premorbid verbal intelligence mean and standard deviation of the mean are reported. For all other variables frequencies are reported stating the number of participants that fulfilled the criteria (i.d. smoking, right handedness, currently on antipsychotic medication).

### 3.3 Ratings of perceived stimulation

Participants of both groups equally often guessed that they had received verum or sham stimulation in session II (*χ2* = 1.34, *p* = 0.25). Ratings of confidence of guessing were the same in both groups (*U* = 279, *Z* = 0.18, *p* = 0.85) as were ratings of pain (*U* = 265.5, *Z* = 0.48, *p* = 0.64). Generally, rating of pain were comparatively low (verum group *M* = 17.17, *SD* = 22.69, sham group *M* = 11.83, *SD* = 16.35, VAS from 0 to 100). Other reported side effects were burning, tingling or itching sensations on the skin, optical effects, a feeling of heat, disturbed concentration or tiredness. However, groups did not differ on their reported side effects (see Table 2 for an overview on reported side effects). Thus, the stimulation was generally well tolerated and participants were not able to discern verum from sham stimulation.

**Table 2:** Overview on side effects during tDCS-stimulation.

	Verum group	Sham group	Total
Burning sensation	7	4	11
Tingling sensation	7	5	12
Itching sensation	2	1	3
Optical effects	2	3	5
Others	3	6	9

frequency of reported side effects of the stimulation separated by group (verum vs. sham) and in total. The number states how many participants of the respective groups (of n = 24 per group) and in total (of N = 48) reported the specific side effect. “Optical effects” included phosphene, blurred vision and tunnel vision, “Others” a feeling of warmth or heat, disturbed concentration, tiredness as well as “a pulling sensation” in the head.

### 3.4. Neuropsychological performance—Main analysis

#### 3.4.1 Baseline differences between groups in session I

No baseline differences were observed for any performance measures of any neuropsychological test (all *p* >.5). Thus, the sham and the verum group did not differ in their baseline performance in session 1.

#### 3.4.2 Effects of the stimulation on neuropsychological performance

**Fig 2:**
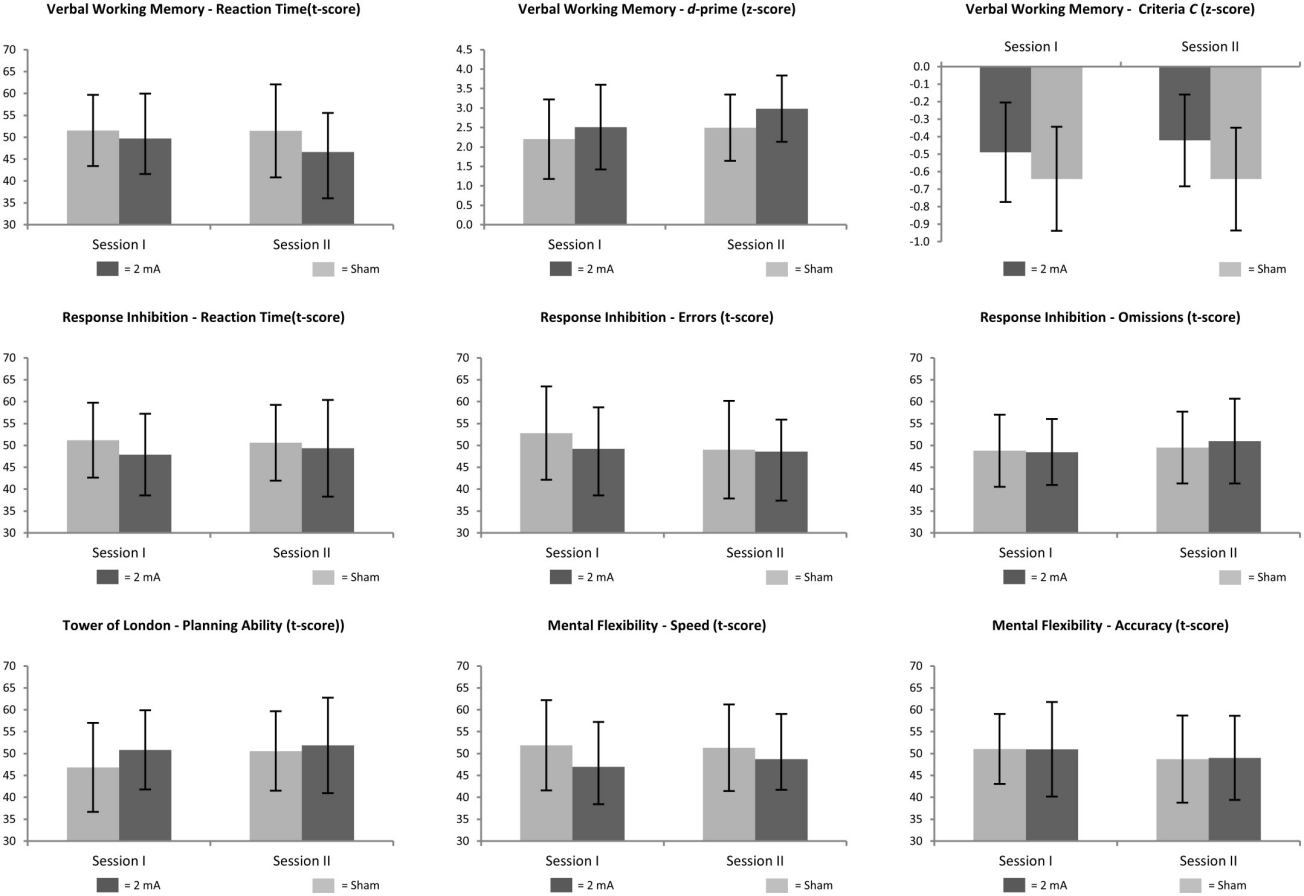
Neuropsychological performance in session 1 and session II separated by group (2 mA verum vs. sham stimulation)—Results of the main analysis. Standardized data (t-scores and z-scores) of neuropsychological performance of the verbal working memory, response inhibition, mental flexibility and planning ability task separated by time of measurement and group (black = 2 mA of tDCS over the left DLPFC, grey = sham condition). Depicted is the respective group mean together with the standard deviation. Note, lower t-scores indicate better performance except for the planning ability task where higher t-scores indicate better performance. For *d*-prime and the response criteria *C* z-scores are presented where higher *d*-prime values indicate better performance and higher *C* values indicate a more conservative response decision.

We observed main effects of TIME in the dependent variables *d-prime* in the verbal working memory task (*F* (1, 46) = 8.85, *p* < 0.01, *η*_*p*_^*2*^ = 0.16), *errors* in the response inhibition task (*F* (1, 46) = 7.03, *p* = 0.01, *η*_*p*_^*2*^ = 0.13), as well as a trend in *problem solving (F* (1, 46) = 3.35, *p* = 0.07, *η*_*p*_^*2*^ = 0.07). Neuropsychological performance in all three performance measures improved from session 1 to session 2. Moreover, a main effect of GROUP was observed in the dependent variable *response criteria C* in the verbal working memory task (*F* (1, 46) = 8.16, *p* = 0.01, *η*_*p*_^*2*^ = 0.15), reflecting a more conservative response criterion in the sham compared to the verum group. A trend wise significance for the TIME*GROUP interaction was reached in the dependent variable *errors* in the response inhibition task (*F* (1, 46) = 3.58, *p* = 0.064, *η*_*p*_^*2*^ = 0.07), indicating a stronger improvement from session 1 to session 2 in the sham compared to the verum group. All other TIME*GROUP interactions in any of the other performance measures in verbal working memory, response inhibition, mental flexibility or problem solving failed to reach significance. Thus, except for the trend in the improvement in errors in the response inhibition task in the sham-group performance of patients in none of the neuropsychological tests performed depended on whether they received sham or verum stimulation in session 2. See Fig 2 for a graphical depiction of the main results and S1 Table for an overview on means and standard deviations and Table 3 for an overview on statistical main effects and interactions.

**Table 3:** Main and interaction effects of different neuropsychological performance measures.

	Main Effect Group			Main Effect Time			Interaction Time*Group		
**Verbal Working Memory**	***F* (1, 46)**	***p***	***η***_***p***_^***2***^	***F* (1, 46)**	***p***	***η***_***p***_^***2***^	***F* (1, 46)**	***p***	***η***_***p***_^***2***^
Reaction Time	1.85	0.18	0.04	1.46	0.23	0.03	1.33	0.25	0.03
*d*-prime	2.67	0.11	0.05	8.85	0.00	0.16	< 1	0.49	0.01
Criteria *C*	8.16	0.016	0.15	< 1	0.51	0.01	< 1	0.49	0.01
**Response Inhibition**									
Reaction Time	< 1	0.40	0.02	0.35	0.55	0.01	1.88	0.18	0.04
Errors	< 1	0.46	0.01	7.03	0.01	0.13	3.58	0.06	0.07
Omissions	< 1	0.79	0.00	2.35	0.13	0.05	< 1	0.41	0.01
**Planning Ability**	1.11	0.30	0.02	3.35	0.07	0.07	1.09	0.30	0.02
**Mental Flexibility**									
Speed	2.63	0.11	0.05	< 1	0.60	0.01	1.04	0.31	0.02
Accuracy	< 1	0.97	0.00	2.03	0.16	0.04	< 1	0.92	0.00

Main and interaction effects separated by dependent variable. *ηp2* = partial eta square. All neuropsychological testing was carried out using the Vienna Test System of Schuhfried, Mödling, Austria, employing the following tests: N-Back Verbal = verbal working memory, INHIB = response inhibition, Tower of London = problem solving, SWITCH = mental flexibility.

### 3.5. Neuropsychological performance—Exploratory analysis

#### 3.5.1 “Online” stimulation

We did not observe an interaction effect TIME*GROUP in any dependent variable in participants who had performed the specific neuropsychological test during “online” stimulation (all *p* >.05, see Table 4). Thus, as for the main analysis, the kind of “online” stimulation received, did not influence performance in the neuropsychological tests.

**Table 4:** Interaction effects TIME*GROUP of different neuropsychological performance measures during “online” vs. “offline” stimulation.

	Online Stimulation			Offline Stimulation		
**Verbal Working Memory**	***F* (1, 22)**	***p***	***η***_***p***_^***2***^	***F* (1, 22)**	***p***	***η***_***p***_^***2***^
Reaction Time	< 1	0.587	0.014	1.235	0.279	0.053
*d*-prime	< 1	0.774	0.004	< 1	0.490	0.022
Criteria *C*	< 1	0.727	0.006	< 1	0.557	0.016
**Response Inhibition**						
Reaction Time	2.234	0.149	0.092	< 1	0.702	0.007
Errors	< 1	0.663	0.009	**5.833**	**0.024**	**0.210**
Omissions	< 1	0.680	0.008	1.959	0.176	0.082
**Planning Ability**	< 1	0.973	0.001	1.631	0.215	0.069
**Mental Flexibility**						
Speed	< 1	0.877	0.001	2.076	0.164	0.086
Accuracy	< 1	0.711	0.006	< 1	0.827	0.002

interaction effects separated by dependent variable and by timing of the testing relative to the current stimulation (i.d. participants that had received the specific test “online” vs. “offline”). *ηp2* = partial eta square. All neuropsychological testing was carried out using the Vienna Test System of Schuhfried, Mödling, Austria, employing the following tests: N-Back Verbal = verbal working memory, INHIB = response inhibition, Tower of London = problem solving, SWITCH = mental flexibility.

#### 3.5.2 “Offline” stimulation

**Fig 3:**
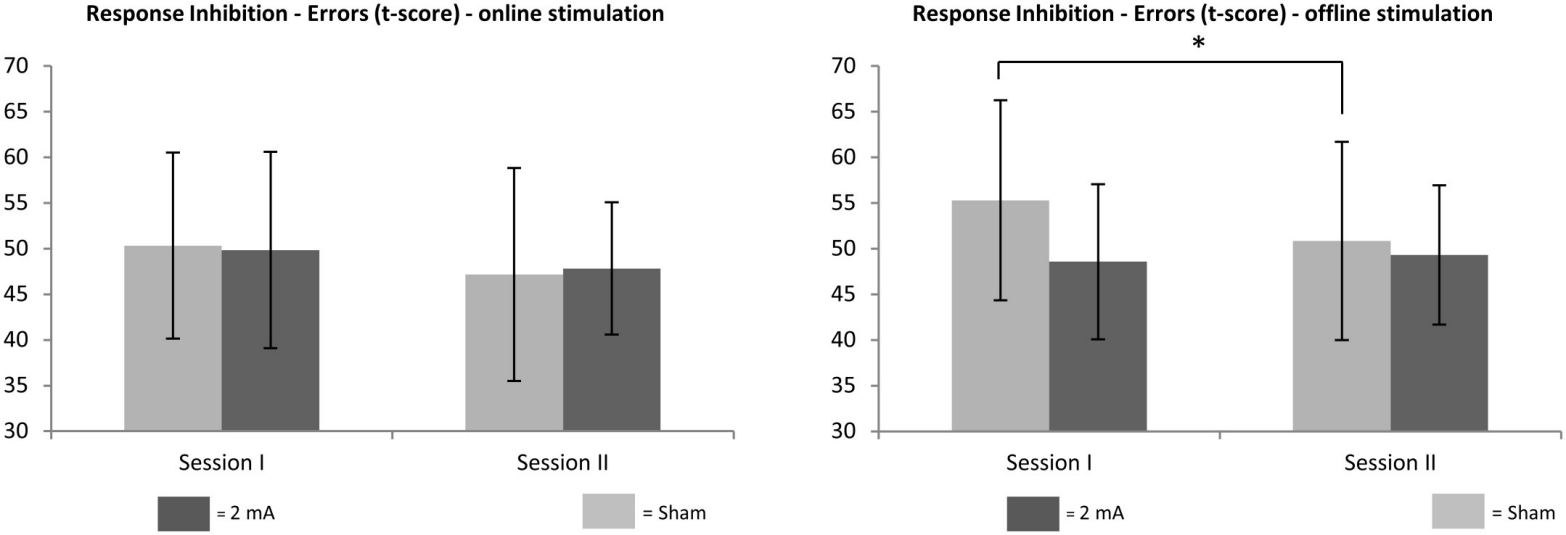
Neuropsychological performance in errors in the response inhibition task: “Online” vs. “offline” results. Standardized data (t-scores) of neuropsychological performance of errors in the response inhibition task separated for patients that engaged in the task during (“online”–left figure) vs. after (“offline”–right figure) stimulation separated by time of measurement and group (black = 2 mA of tDCS over the left DLPFC, grey = sham condition). Depicted is the respective group mean together with the standard deviation. Lower t-scores indicate better performance. * = significant interaction effect TIME*GROUP.

A TIME*GROUP interaction was observed in the dependent variable *errors* in the response inhibition task (*F* (1, 22) = 5.83, *p* = 0.024, *η*_*p*_^*2*^ = 0.21), indicating a stronger improvement from session 1 to session 2 in the sham compared to the verum group in participants who had performed the neuropsychological response inhibition task within 20 minutes after the end of the stimulation (see Fig 3 for a graphical depiction). All other TIME*GROUP interaction in any other dependent variable failed to reach significance (all *p* >.05, see Table 4). Thus, patients who received a verum stimulation showed an impaired performance in the dependent variable *errors* in the response inhibition task within 20 minutes after the end of the stimulation compared to participants who received a sham stimulation.

## 4. Discussion

Recent research suggests tDCS to enhance cognitive functions in patients with schizophrenia. The question regarding its acute effects on executive functions, however, remains largely unanswered. Following Miyake´s taxonomy of executive functions [7, 8] we examined the influence of 20 minutes of 2 mA of tDCS applied over the left DLPFC on performance in verbal working memory (updating), response inhibition (inhibition), mental flexibility (shifting) and problem solving. Patients with schizophrenia, schizoaffective disorder or acute transient psychotic disorder were tested twice according to a randomized, double blind, sham-controlled repeated-measures design.

Patients of both groups were not able to correctly discriminate the type of stimulation received confirming the success of the blinding procedure. However, in the main analysis of the whole sample the change in performance from session 1 to session 2 was the same in the verum as in the sham group. Moreover, in an additional exploratory analysis, neuropsychological performance during “online” stimulation was also the same irrespective of whether patients received a sham or a verum stimulation. However, the exploratory analysis for the “offline” group also demonstrated that the improvement in errors in the response inhibition task from session I to session II was stronger in patients who engaged in the task within 20 minutes after the end of the sham stimulation compared to those who had received a verum stimulation, suggesting an inhibitory effect of the verum stimulation on task performance. Thus, using the above stated stimulation settings of tDCS and the specific neuropsychological tests described, we did not find an acute enhancing effect of tDCS on executive functions in our patient group, but an impaired function in one task shortly after.

Our study has several advantages, which supports us to draw clinically valid conclusions from its outcomes. First, we adopted a double blind, sham controlled design. This allowed us to assess tDCS effects independently of the expectations of both patients or experimenter. Patients were randomly assigned to either verum or sham stimulation during the second session and neither they nor the performing experimenter were aware of the type of stimulation. Our blinding was successful as evidenced by the fact that patients in both groups did not differ in their guessing concerning the type of stimulation they received or in their self-reported pain inflicted by the stimulation. Thus, our manipulation was sustainable and we can assume that effects of expectancy such as placebo or nocebo did not influence the outcome. Secondly, the clinical sample investigated was not preselected but represented a typical clinical sample of inpatients with schizophrenia spectrum of our institutions. This emphasizes the clinical generalizability of our results. Thirdly, the neuropsychological assessments used in the current study were psychometrically well constructed and clinically validated tests commonly employed in a vast number of psychiatric and neurological hospitals worldwide. Hence, if tDCS affected performance in these tests it should have a high external validity and be of practical relevance outside from the laboratory setting. Lastly, by applying a repeated-measures design we were able to statistically take into account preexisting baseline differences between both groups, which might have biased the results. Moreover, it increased our statistical power and allowed us to detect clinically meaningful effect size of *d* = 0.5 [41] with a high probability.

Patients of both groups did not differ in their change in working memory performance from session 1 to session 2, irrespective of whether “online” and “offline” effects were analyzed together or whether “online” and “offline” effects were analyzed separately. This is compatible with previous studies using the same stimulation configuration that either reported an improvement in *d-prime* only following an “online” stimulation of 1 mA but not 2 mA [25], or only after 20 to 40 minutes but not directly after stimulation of 2 mA [26]. Interestingly, a recent combined behavioral and neuroimaging study [28] showed that 2 mA of anodal tDCS applied over F3 acutely enhanced the BOLD-signal in the medial frontal cortex but did not acutely influence working memory performance. Moreover, in this study, beneficial effects of tDCS appeared 24 h after stimulation. Our and the previous results suggest intensity and time specific effects of tDCS on working memory with an advantage of 1 mA over 2 mA during only stimulation and beneficial effects of 2 mA to appear with a sufficient time interval relative to stimulation only.

In our experiment, tDCS did not beneficially affect neuropsychological performance in response inhibition. Contrary, the number of errors in the response inhibition task decreased over time in the sham compared to the verum group at a trend level significance if “online” and “offline” effects were analyzed together. Moreover and importantly, a subgroup analysis comparing the performance of patients that received verum or sham stimulation either within the first 20 minutes of the experiment (i.e. “online”) or within the second 20 minutes (i.e. “offline”) further disentangled this effect: Patients that engaged in the response inhibition task “offline” and had received a verum stimulation in the first 20 minutes showed less improvement from session I to session II in errors committed in the response inhibition task compared to patients who had received a sham stimulation. This indeed, suggests a detrimental effect of 2 mA of tDCS applied over the left DLPFC on response inhibition that emerges shortly after the end of the stimulation. As response inhibition is generally considered to be a cognitive function that is neuroanatomically largely based on the right inferior frontal cortex [31], our results suggest that the placement of the cathode electrode on FP2 might have exerted an excitatory diminishing effect that ultimately impaired response inhibition performance. Interestingly, detrimental effects of the same stimulation configuration of tDCS in patients with schizophrenia were recently also shown for performance in visual attention albeit 24 h after stimulation [42], as well as on verbal working memory within 100 minutes after stimulation [27]. Taken together these findings suggest that tDCS does not essentially always improve cognition but might also further impair cognitive functions in patients with schizophrenia depending on electrode configuration and placement.

To the best of our knowledge, only two other studies investigated tDCS effects on executive functions in patients with schizophrenia beyond working memory so far. Rassovsky and colleagues [27] investigated the effects of 2 mA of anodal tDCS applied over the left DLPFC and report no effects on problem solving. Given that the authors do not clearly state which neuropsychological test they applied, is it difficult to compare their finding to ours. However, neither their nor our finding suggest an acute enhancing effect of anodal tDCS over F3 on problem solving. However, the previous and our finding might need to be interpreted within a recent theoretical framework that divides executive functions into “hot” executive functions related to reward, emotion and motivation such as emotion regulation, delay discounting and risky and affective decision making and purely, cognitive “cold” executive functions such as working memory, response inhibition, cognitive flexibility and planning ability [43]. As problem solving and planning ability have in healthy participants recently been shown to not be a purely “cold” but also to a certain extent a “hot” executive functions, it is possible that our stimulation parameters were not optimal for the Tower of London task [44]. Orlov and colleagues [28] report an acute improvement in accuracy in the Stroop task during “online” stimulation with 2 mA at F3. Contrary, we did not observe an effect of tDCS in the mental flexibility task that we employed. Possibly, differences in task design between the two neuropsychological tests explain this discrepancy.

We need to acknowledge several limitations: First, in the statistically fully powered main analysis for which the experiment was initially conceived our design does not allow disentangling “online” from “offline” effects. To avoid effects of sequence we counterbalanced the order of presentation of the neuropsychological test. Given that the duration of the stimulation was less than the time needed to perform the neuropsychological tests each test was both presented during or shortly after the stimulation. Thus, each test was presented both “online” and “offline”, albeit in different individuals. However, given that the stimulation ran for 20 minutes and the execution of the tests took not more than 40 minutes the offline time interval was comparably short (i.e. not more than 20 minutes) and most tests were either presented “online” or shortly afterwards. Since a recent study confirmed that after effects of different intensities of tDCS including 2 mA last for at least 30 minutes [24], we assumed that the effects of the stimulation were still in place during the last neuropsychological test. Thus, we assumed that we investigated the acute effects of an active stimulation, even if during some of the task the direct current flow had already stopped. Our subsequent, less-powered, exploratory analysis largely confirmed the results of our main analysis, showing that for verbal working memory, mental flexibility as well as for planning ability the results were the same irrespective of whether a whole sample analysis was conducted or whether separate analysis for “online” vs. “offline” effects were performed. However, we did find a difference in errors the response inhibition task, showing that the detrimental effects of the verum stimulation only appeared shortly after the end of the stimulation, i.d. during the “offline” condition. Secondly, while we asked patients for their current smoking status, we did not control whether patients smoked shortly before the experiment or not. However, both groups included the same number of smokers vs. non-smokers. Third, our sample was not fully homogenous and, while largely comprising patients with a diagnosis of schizophrenia (ICD-10: F20.0) it also included patients with schizoaffective disorder (ICD-10: F25) or acute transient psychotic disorder (ICD-10: F23). This heterogeneity might have influenced the results. Fourth, data of handedness and duration of disease was not available for all patients. However, as the available data showed no differences between the two groups on these parameters we are confident that both groups were fully comparable. Fifth and lastly, we need to acknowledge that after all the missing of a significant effect does not imply that there were no differences. Although we *a priori* calculated our sample size for a clinically meaningful effect of *f = 0*.*25* we cannot rule out that there were smaller differences between both groups which we were not able to statistically prove, or that some characteristics of our sample (such as the variability of the diagnostic groups) made it difficult to find an effect. Thus, there remains an uncertainty concerning the acute effects of our stimulation parameters of tDCS on executive functions in patients with schizophrenia or related disorders which future studies with larger populations might be able to resolve.

## 5. Conclusion

In summary, in the current experiment 2 mA of tDCS applied for 20 minutes over the left DLPFC at F3 (cathode at FP2, supraorbital ridge) did not acutely improve neuropsychological performance in several executive functions, namely verbal working memory, response inhibition, mental flexibility and problem solving in patients with schizophrenia and related disorders. Moreover, a detrimental effect on response inhibition shortly after the end of stimulation was observed for patients that had received a verum stimulation. Future studies should continue to seek for the most effective and beneficial stimulation settings of tDCS for this patient group.

## Supporting information

S1 Fig Graphical depiction of the four neuropsychological tests used in the study. Graphical depiction of the four neuropsychological tests used in the study. A: Tol = Tower of London task used to assess planning ability. B: NBV = N-back verbal, verbal working memory task used to assess verbal working memory performance. C: INHIB: Response inhibition task used to assess response inhibition performance. D: SWITCH, switching task used to assess shifting and switching ability. See also methods section for a more detailed description.
(TIF)

S1 Table Standardized data of neuropsychological performances measures. Standardized data (t-scores and z-scores) of neuropsychological performance measures separated by time of measurement and group. Note, lower t-scores indicate better performance except for problem solving where higher values indicate better performance. For *d*-prime and the response criteria *C* z-scores are presented where higher *d*-prime values indicate better performance and higher *C* values indicate a more conservative response decision.
(DOCX)

S1 File Consort checklist. CONSORT 2010 checklist of information to include when reporting a randomised trial.
(DOC)

S2 File Ethical report English version. Report to the Ethics Committee English Version.
(DOC)

S3 File Ethical report German version. Report to the Ethics Committee German Version.
(DOC)
