# Supplementary figures and images for: Acute effects of a single dose of 2 mA of anodal transcranial direct current stimulation over the left dorsolateral prefrontal cortex on executive functions in patients with schizophrenia—A randomized controlled trial
Source: PLoS One. 2021 Jul 16;16(7):e0254695. doi: 10.1371/journal.pone.0254695 (PMC8284793; doi:10.1371/journal.pone.0254695)

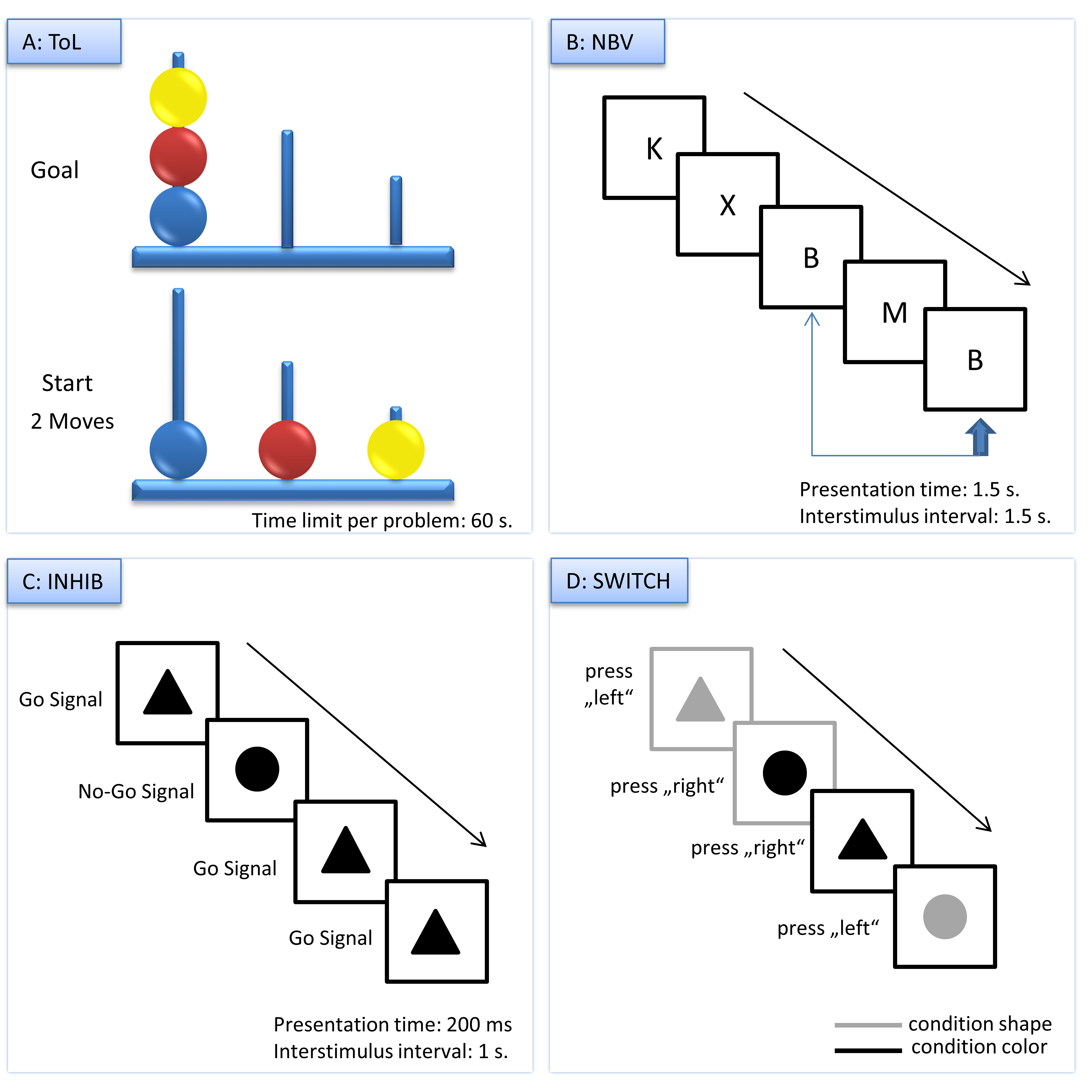

Supplement: S1 Fig — Graphical depiction of the four neuropsychological tests used in the study. A: Tol = Tower of London task used to assess planning ability. B: NBV = N-back verbal, verbal working memory task used to assess verbal working memory performance. C: INHIB: Response inhibition task used to assess response inhibition performance. D: SWITCH, switching task used to assess shifting and switching ability. See also methods section for a more detailed description. (TIF) [file pone.0254695.s001.tif]
