# Supplementary material for: Acute effects of a single dose of 2 mA of anodal transcranial direct current stimulation over the left dorsolateral prefrontal cortex on executive functions in patients with schizophrenia—A randomized controlled trial
Source: PLoS One. 2021 Jul 16;16(7):e0254695. doi: 10.1371/journal.pone.0254695 (PMC8284793; doi:10.1371/journal.pone.0254695)
Supplement: S1 Table — Standardized data (t-scores and z-scores) of neuropsychological performance measures separated by time of measurement and group. Note, lower t-scores indicate better performance except for problem solving where higher values indicate better performance. For d-prime and the response criteria C z-scores are presented where higher d-prime values indicate better performance and higher C values indicate a more conservative response decision. (DOCX) [file pone.0254695.s002.docx]

**S1 Table: standardized data of neuropsychological performances measures**

|  |  | **First Session(Baseline)** | |  | **Second Session (Stimulation)** | | | **First Session (Baseline)** | | | **Second Session(Stimulation)** | | |
| --- | --- | --- | --- | --- | --- | --- | --- | --- | --- | --- | --- | --- | --- |
| **Neuropsychological test** |  | **Verum Group** | |  | **Verum Group** | |  | **Sham Group** | |  | **Sham Group** | |  |
|  |  |  |  |  |  |  |  |  |  |  |  |  |  |
| **Verbal Working Memory** |  | **Mean** | **SD** |  | **Mean** | **SD** |  | **Mean** | **SD** |  | **Mean** | **SD** |  |
| Reaction Time (t-score) |  | 49.72 | 10.21 |  | 46.65 | 8.92 |  | 51.51 | 8.14 |  | 51.44 | 10.65 |  |
| *d*-prime (z-score) |  | 2.51 | 1.09 |  | 2.98 | 0.85 |  | 2.20 | 1.02 |  | 2.49 | 0.85 |  |
| Criteria *C* (z-score) |  | -0.49 | 0.28 |  | -0.42 | 0.26 |  | -0.64 | 0.30 |  | -0.64 | 0.29 |  |
|  |  |  |  |  |  |  |  |  |  |  |  |  |  |
| **Response Inhibition** |  |  |  |  |  |  |  |  |  |  |  |  |  |
| Reaction Time (t-score) |  | 47.90 | 9.32 |  | 49.33 | 11.05 |  | 51.16 | 8.56 |  | 50.59 | 8.68 |  |
| Errors (t-score) |  | 49.21 | 9.51 |  | 48.57 | 7.32 |  | 52.81 | 10.66 |  | 49.00 | 11.17 |  |
| Omissions (t-score) |  | 48.47 | 7.55 |  | 50.97 | 9.65 |  | 48.77 | 8.25 |  | 49.52 | 8.20 |  |
|  |  |  |  |  |  |  |  |  |  |  |  |  |  |
| **Problem solving (t-score)** |  | 50.84 | 9.04 |  | 51.87 | 10.91 |  | 46.82 | 10.16 |  | 50.57 | 9.09 |  |
|  |  |  |  |  |  |  |  |  |  |  |  |  |  |
| **Mental Flexibility** |  |  |  |  |  |  |  |  |  |  |  |  |  |
| Speed (t-score) |  | 46.94 | 8.50 |  | 48.73 | 6.99 |  | 51.90 | 10.31 |  | 51.33 | 9.89 |  |
| Accuracy (t-score) |  | 50.98 | 10.83 |  | 48.98 | 9.60 |  | 51.06 | 7.99 |  | 48.74 | 9.98 |  |

Table II: standardized data (t-scores and z-scores) of neuropsychological performance measures separated by time of measurement and group. Note, lower t-scores indicate better performance except for problem solving where higher values indicate better performance. For *d*-prime and the response criteria *C* z-scores are presented where higher *d*-prime values indicate better performance and higher *C* values indicate a more conservative response decision.
