# Supplementary material for: Acute effects of a single dose of 2 mA of anodal transcranial direct current stimulation over the left dorsolateral prefrontal cortex on executive functions in patients with schizophrenia—A randomized controlled trial
Source: PLoS One. 2021 Jul 16;16(7):e0254695. doi: 10.1371/journal.pone.0254695 (PMC8284793; doi:10.1371/journal.pone.0254695)
Supplement: S2 File — Report to the Ethics Committee English Version. (DOC) [file pone.0254695.s004.doc]

# 1. Cover page

Project title: Acute effects of transcranial direct current stimulation on executive function disorders in patients with schizophrenia

Principal investigator: Assoc. Prof.Matthias Weisbrod (med.)

Co-director of the Neurocognition Working Group

Clinic for General Psychiatry

Heidelberg University Hospital

Chief physician

Department of Psychiatry and Psychotherapy

SRH Clinic Karlsbad-Langensteinbach

Phone: 07202-613342

Email: [matthias.weisbrod@srh.de](mailto:matthias.weisbrod@srh.de)

Other contributors: Dr. Steffen Aschenbrenner (PhD, Dipl. Psych.)

Senior Psychologist

Department of Clinical and Neuropsychology

SRH Clinic Karlsbad-Langensteinbach

Phone: 07202-613602

Email: [steffen.aschenbrenner@srh.de](mailto:steffen.aschenbrenner@srh.de)

Dr. Thomas Schilling (PhD natural science, Dipl. Psych.)

Department of Clinical and Neuropsychology

SRH Clinic Karlsbad-Langensteinbach

Phone: 07202-613058

Email: [thomas.schilling@srh.de](mailto:thomas.schilling@srh.de)

Biometricians: The study itself does not involve a biometrician, nor will a biometrician be consulted during the planning or the statistical analysis of the study.

Funding: The study receives financial backing from the 'SRH Förderstiftung' (the SRH Holding Foundation)

Date/Version : 5th July 2018/ Version_3

Signature of the Principal Investigator

Assoc. Prof. M. Weisbrod (med.)

# 2. Summary

Cognitive disorders such as deficiencies in executive functions are frequently observed in patients with schizophrenia and are the most significant predictor for restoring or maintaining the ability to work. The treatment of these disorders is therefore of great importance. A promising novel therapeutic option is the neuromodulatory approach of transcranial direct current stimulation. The aim of the proposed study submitted for review is to investigate short-term effects of direct current stimulation on executive function disorders in patients with schizophrenia.

Study design: Multivariate, two-factor mixed-model design with two-tiered between-subject factor *groups* (verum stimulation vs. sham stimulation) and two-tiered within-subject factor *time* intervals (pre vs. post). Patients undergo several neuropsychological procedures before and after the stimulation to assess executive functions.

Patient population: A total of 50 patients with an F2.x diagnosis (i.e. schizophrenia, schizotypal disorder, delusional disorder or schizoaffective disorder) will be randomly assigned to two groups of 25 patients. The proposed study will not involve initiating patient medical drug treatments, nor will the proposed study change any existing medical drug treatments taken (e.g. dose changes). The total time commitment for patients wishing to participate in the study is approx. 60 minutes for inpatients and approx. 3 hours for outpatients.

The study will be conducted in accordance with the provisions of the Professional Code of Conduct for Physicians and in accordance with the current version of the 2013 Helsinki Declaration. Patients will be given written and verbal information before the start of the study and will provide their informed consent in writing. A patient can withdraw their informed consent at any time without providing any justification. Transcranial direct current stimulation is risk free and has few side effects when applied correctly.

**Table of contents**

[1. Cover page 1](#__RefHeading___Toc47961072)

[2. Summary 3](#__RefHeading___Toc47961073)

[3. Introduction/Scientific principles 6](#__RefHeading___Toc47961074)

[3.1. Executive functions 6](#__RefHeading___Toc47961075)

[3.2 Cognitive disorders in patients with schizophrenia 7](#__RefHeading___Toc47961076)

[3.3 Transcranial direct current stimulation 8](#__RefHeading___Toc47961077)

[4. Objective of the study 10](#__RefHeading___Toc47961078)

[5. Primary and secondary endpoints 11](#__RefHeading___Toc47961079)

[6. Study related procedures/study timeline 11](#__RefHeading___Toc47961080)

[6.1 Recruitment of patient population 11](#__RefHeading___Toc47961081)

[6.2 Informing the probands 12](#__RefHeading___Toc47961082)

[6.3 Collection of psychometric and clinical data 12](#__RefHeading___Toc47961083)

[6.4 Standardised and normalised neuropsychological test procedures to assess cognitive performance: 12](#__RefHeading___Toc47961084)

[6.4 Stimulation protocol 14](#__RefHeading___Toc47961085)

[6.5 Experimental procedure and time demands on the patients 14](#__RefHeading___Toc47961086)

[6.6 Data protection policy 15](#__RefHeading___Toc47961087)

[6.7 Intended start and expected completion of the study 15](#__RefHeading___Toc47961088)

[7. Effect/Benefits 15](#__RefHeading___Toc47961089)

[8. Time commitment/risks 15](#__RefHeading___Toc47961090)

[9. Study design 16](#__RefHeading___Toc47961091)

[10. Inclusion/exclusion criteria 16](#__RefHeading___Toc47961092)

[11. Criteria for withdrawal 17](#__RefHeading___Toc47961093)

[11.1 Individual criteria for withdrawal 17](#__RefHeading___Toc47961094)

[11. 2 Criteria for withdrawal of the entire study 17](#__RefHeading___Toc47961095)

[12. Randomisation procedure 17](#__RefHeading___Toc47961096)

[13. Statistical design 17](#__RefHeading___Toc47961097)

[13.1 Proposed size of the patient population 18](#__RefHeading___Toc47961098)

[13.2 Statistical analysis 18](#__RefHeading___Toc47961099)

[13. Legal and ethical considerations 19](#__RefHeading___Toc47961100)

[14. Funding, immaterial support, institutional affiliations, potential conflicts of interest, incentives for participants, compensation 20](#__RefHeading___Toc47961101)

# 3. Introduction/Scientific principles

Schizophrenia is a heterogeneous psychiatric disorder, often accompanied by massive limitations on the ability to participate in social life and to lead an independent life. In addition to striking clinical symptoms such as delusion, changes in perception, thought disorders and avolition, patients often suffer from cognitive impairment such as deficiencies in executive functions. Executive functions are the best long-term predictors for maintaining or regaining everyday functions and their restoration is therefore paramount. Modern neuroscientific stimulation approaches such as transcranial direct current stimulation may significantly contribute to achieving this objective. The study submitted for review will investigate the short-term effects of this approach on executive function disorders in patients with schizophrenia. The relevant theoretical background is presented below.

## 3.1. Executive functions

In neuropsychology, the term executive functions refers to cognitive control and management functions. According to Müller (2013) these are “regulation and control mechanisms that facilitate goal-oriented and situation-adapted behaviour” as well as metacognitive processes that serve to achieve a defined goal as well as to develop new goals. They are characterised by both flexibility in the sense of adaptation to changing requirements and persistence, i.e. the pursuit of a goal and isolation of an action from distractions (Müller, 2013). Executive functions enable an organism to act in a goal-oriented and flexible manner, especially when routines are no longer sufficient to solve a problem.

Most definitions of executive functions share the common feature that they involve several cognitive processes. Miyake et al. (2000) for example differentiate three basic mechanisms: 1). Shifting (changing the focus of attention), 2). Updating (updating working memory content) and 3). Inhibition (suppression of dominant response patterns). The model specifies that a combination of all three basic mechanisms are required to generate a complex executive function. In a clinical definition of executive functions, Müller et al. (2004) differentiate between the following domains 1) working memory and monitoring, 2). cognitive flexibility and 3). problem solving. In line with these definitions, executive functions are typically assessed and measured in everyday clinical practice using tasks involving working memory, flexibility and shifting, response inhibition and planning.

In terms of neuroanatomy, executive functions are specifically associated with the prefrontal cortex. Numerous clinical case reports and research studies have shown that damage to this area is associated with deficiencies in planning, working memory, response inhibition, flexibility and persistence (Müller, 2013). According to Cicerone et al. (2006) it is possible to assign different executive functions to distinct neuroanatomical regions. Cognitive functions such as planning, monitoring, shifting, working memory and inhibition are thus mediated by the dorsolateral prefrontal cortex (DLPFC). An organism's ability to self-regulate is closely linked to the ventromedial prefrontal cortex, motivation and stimulation to the mediofrontal cortex and metacognitive processes to the frontal poles.

Executive function disorders occur in a variety of neurological and psychiatric diseases. One patient population that often suffers from executive function deficiencies are patients with schizophrenia.

## 3.2 Cognitive disorders in patients with schizophrenia

Schizophrenia is a heterogeneous psychiatric disease with a worldwide prevalence of approximately 1%. Clinical characteristics include positive symptoms (delusion, paranoia and hallucinations) and negative symptoms (reduced affect display, apathy, lack of willpower, poverty of ideas, catatonia) as well as disorganised behaviours (inappropriate affect, absentmindedness, confusion) (Fisher, Herman, Stephens & Vinogradov, 2016; Leucht, Vauth, Olbrich & Jäger, 2014).

In addition to these main symptoms, it has been observed for over a century that patients with schizophrenia suffer from cognitive disorders. Kraepelin for example referred to *dementia praecox* and first described a dementia-related deterioration beginning in early adulthood with various cognitive impairments as one of the characteristic symptoms of the disease. Epidemiological studies indicate that cognitive disorders occur in up to 80% of all patients (Leucht et al., 2014). These manifest as both a reduction in the general level of intelligence ('IQ') and the impairment of a large number of cognitive partial functions: predominantly affecting attention, memory and executive functions (for an overview see Exner & Lincoln, 2012). In the case of memory disorders, the extent of verbal learning and memorisation impairment is striking. Attention disorders include impairment of processing speed, vigilance and selective attention. Deficiencies in executive functions result in impairment of verbal working memory, problem solving and cognitive flexibility (e.g. Dickinson, Ramsey & Gold, 2007; Mesholam-Gately, Giuliano, Goff, Faraone & Seidman, 2009).

Deficiencies in executive functions suggest that prefrontal structures are involved in the pathophysiology of cognitive disorders in patients with schizophrenia. This was indeed confirmed by imaging studies. Patients with schizophrenia exhibit reduced brain volume compared to healthy controls, including in the dorsolateral prefrontal cortex and also in the medial temporal lobe and other brain regions (Glahn et al., 2008). Functional imaging studies (PET, fMRI) have also demonstrated reduced metabolic activity in the DLPFC region. This is evident both in the resting state and during task processing which places additional demands on executive functions particularly on the left hemisphere (Minzenberg, Laird, Thelen, Carter & Glahn, 2009).

During the acute phase of the disease, positive symptoms and disorganised behaviour predominate, whereas negative symptoms and cognitive disorders are more prominent in the sub-acute and chronic phases. Cognitive disorders are largely independent of the course of acute symptoms and are stable over time (Harvey, Green, Bowie & Loebel, 2006). Cognitive disorders are particularly relevant for long-term successful vocational rehabilitation and reintegration and represent the most significant prognostic predictors (Nuechterlein et al., 2011; Tsang, Leung, Chung, Bell & Cheung, 2010). Executive functions as well as verbal learning and memorisation are particularly important in this respect (Leucht et al., 2014). The current standard in the modern therapy for patients with schizophrenia therefore focuses on the rehabilitation of cognitive disorders once clinical stabilisation has been achieved. A new therapeutic strategy with significant clinical potential is the application of neuromodulatory techniques such as transcranial Direct Current Stimulation (tDCS).

## 3.3 Transcranial direct current stimulation

Transcranial direct current stimulation is an interventional neurophysiological procedure. The treatment generally involves placing two electrodes on the patient's scalp and then applying a weak direct current from the positive surface anode to the negative surface cathode. It aims to modulate neuronal activity in the targeted brain regions by means of excitation under the anode and inhibition under the cathode (Bodatsch, 2014).

tDCS induces distinct short-term and long-term effects. Short-term effects appear to be mediated by modulation of the spontaneous neuronal network activity during stimulation ('online effects'), resulting in a polarity-dependent shift of the resting membrane potential. Neurons are depolarised under the anode, while the reverse effect occurs under the cathode (Nitsche & Paulus, 2000, 2001). Longer-term effects that go beyond the actual duration of stimulation ('offline effects') are most likely based on similar mechanisms as long-term potentiation or long-term depression, and are glutamatergic. Alterations in neuronal excitability appear up to 60 minutes after the end of stimulation (for an overview see Bodatsch, 2014)

As far as is currently understood, tDCS is a safe, well tolerated neuromodulatory technique with few side effects (Bikson et al., 2016). Potential transient side effects include itching, tingling, headache, burning sensation of the skin, discomfort, phosphenes (short flashes of light), fatigue, nausea, vomiting or temporary dizziness. For research purposes, modern tDCS devices offer the possibility of double-blind placebo stimulation in addition to real verum stimulation. This allows non-specific effects, e.g. effects induced by the practitioner, to be separated from specific effects caused by the stimulation.

### 3.3.1 tDCS and cognition in the healthy population

The effect of transcranial direct current stimulation on cognitive processes has been extensively investigated in healthy probands. Particular focus is placed on verbal working memory, which due to neuroanatomical considerations typically involves anodal stimulation over the left DLPFC. Although results are heterogeneous, they are nevertheless generally positive in terms of improving the performance of working memory (for an overview see Brunoni & Vanderhasselt, 2014; Hill, Fitzgerald & Hoy, 2016). Fregni et al. (2005) for instance demonstrated that anodal tDCS over the left DLPFC improves performance during an N-back working memory task. Pope et al. (2015) showed improved performance using the same stimulation configuration in a demanding but not a simple working memory task. In addition to working memory performance, the effect of tDCS on other executive functions in healthy individuals was also investigated. For example, differential beneficial effects of anodal and cathodal tDCS over the left DLPFC on performance were observed in the ”Tower of London” planning task (Dockery, Hueckel-Weng, Birbaumer & Plewnia, 2009).

### 3.3.2 tDCS and cognition in patients with schizophrenia

The use of transcranial direct current stimulation for the treatment of cognitive deficiencies in patients with schizophrenia has not been widely investigated. Due to the frequently described cerebral hypofrontality, a potential treatment strategy may consist of anodal stimulation over the left DLPFC (for an overview see e.g. Mervis, Capizzi, Boroda & MacDonald, 2017).

In the first study using a single stimulation, Hoy and colleagues (2014) showed that a dose of 2 milliamperes (mA) applied for 20 minutes over the left DLPFC improved performance in a verbal working memory task. Further studies using multiple stimulations were able to confirm this beneficial effect. Smith et al. (2015) for instance observed an improvement in verbal working memory and attentional functions after stimulations to the left DLPFC - again 2 mA applied for 20 minutes - but no effects on reasoning/problem solving or learning ability. The pilot study of Nienow et al. (2016) revealed that simultaneous application of tDCS during working memory training can improve the effect of the training.

In summary, all the above studies provide evidence which supports that tDCS can improve cognitive functions in healthy individuals and in patients with schizophrenia. Although to date numerous studies have reported beneficial effects on verbal working memory, none have as yet demonstrated any effect on other areas of executive functions such as planning ability or response inhibition. This highlights the need for further research to improve existing stimulation protocols and, to ultimately, develop differential therapeutic indications.

# 4. Objective of the study

The primary objective of the proposed research project is to determine whether and to what extent deficiencies in executive functions in patients with schizophrenia can be improved by transcranial direct current stimulation. The project will specifically investigate whether a single tDCS stimulation has short-term, positive effects on executive function disorders in this patient group.

Hypothesis: A single, 2 mA, anodal transcranial direct current stimulation applied over the left prefrontal cortex for a period of 20 minutes leads to short-term improvement of executive function disorders in patients with schizophrenia.

# 5. Primary and secondary endpoints

The primary endpoint for the aforementioned hypotheses is the improvement of cognitive performance in the area of executive functions in patients with schizophrenia, measured using several standardised neuropsychological testing procedures. There are no other secondary objectives.

# 6. Study related procedures/study timeline

## 6.1 Recruitment of patient population

The study will be conducted in the Department of Psychiatry and Psychotherapy at the Karlsbad-Langensteinbach Clinic in Germany. The patient population will be recruited through the wards. Patients will be invited to participate in the study during the routine admission diagnostics assessment. During this first consultation, the patient's inclusion/exclusion criteria will be reviewed and patients will be provided with information about the study. Should the patient give his/her consent, an appointment is then scheduled for the examination *per se*.

In addition to hospitalised patients, we will also recruit from out-patients All patients interested in participating in the study will first be invited to an initial interview, during which the patients' inclusion/exclusion criteria will be reviewed and they will be provided with information about the study. If patients wish to participate in the study, they will undergo some psychological tests and fill in several questionnaires after the interview. These are the same procedures that in-patients also undergo as part of routine clinical diagnostics. An appointment for the actual examination is then arranged to proceed with the stimulation protocol using the tDCS device.

The proposed study will not involve initiating patient medical drug treatments, nor will the proposed study change any existing medical drug treatments taken (e.g. dose changes). Participation in the study neither requires a change of medication nor will it impede a clinically necessary modification.

## 6.2 Informing the probands

Patients will be informed both verbally and in writing prior to the start of the study about the nature and scope of the planned investigation, in particular about the potential benefits to their health and any risks involved. The patient gives his/her informed consent by signing the declaration of consent. Should the patient withdraw from the study, any previously obtained material will be destroyed. Alternatively, the patient may be asked whether he/she agrees to the use of their data in the analysis. Patients' participation in the study is voluntary; consent may be withdrawn at any time without providing any justification and without prejudice to further medical care.

## 6.3 Collection of psychometric and clinical data

To verify the diagnosis, a structured interview (SCID-I and -II or short forms thereof) will be carried out. Further, questionnaires and a number of neuropsychological test procedures will also be implemented. The assessment includes the following procedures:

## 6.4 Standardised and normalised neuropsychological test procedures to assess cognitive performance:

All patients will undergo the COGBAT® test set (Aschenbrenner, Kaiser, Pfüller, Roesch-Ely & Weisbrod, 2012) as part of routine clinical diagnostics or during an additional outpatient consultation before beginning the examination. This test set includes a compilation of key neuropsychological parameters to assess the cognitive status of patients with mental disorders. It covers the following parameters: attention, memory, executive functions and processing speed. Specific focus will be placed on performance in the subtests addressing planning ability (“Tower of London”), verbal working memory (“Nback-Verbal“) and response inhibition (“INHIB”). The “SWITCH” test from the Vienna Test System will also be used to assess cognitive flexibility. See Table 1 for an overview of the methods used.

Table 1: Overview of diagnostic, therapeutic and interventional procedures

| **Clinical diagnostics and psychopathology**   - Sociodemographic interview - SCID-I (Wittchen, Zaudig & Fydrich, 1997) - SCID-II - Montgomery-Asberg Depression Rating Scale (MADRS, Montgomery & Asberg, 1979) - Beck Depression Inventory (BDI-II, Hautzinger, Keller & Kühner, 2006) - Personality style and disorder inventory (PSSI, Kuhl & Kazén, 2009) - Inventory of social competence (ISK, Kanning, 2009) - Emotional Competence Questionnaire (EKFS, Rindermann, 2009) - Cognitive performance questionnaire* (FLei, Beblo, Kunz, Lautenbacher, Albert & Aschenbrenner, 2011) |
| --- |
| **Premorbid intelligence**   - MWT-B (Lehrl, 2005) (German multiple choice word selection test) |
| **Neuropsychological diagnostic procedures**  **Vienna test system**  *Information processing speed*   - Trail Making Test Langensteinbach Version*   *Attention*   - Divided attention WAFG test* - Alertness-WAFA test*   *Memory*   - FGT (Figural Memory Test)*   *Executive functions*   - Working memory N-back verbal* - Inhibition-INHIB Go-Nogo* - Cognitive Flexibility Trail Making Test Langensteinbach Version* - Cognitive Flexibility- SWITCH - Convergent Planning-Tower of London-Freiburg Version* |
| **Neurophysiological stimulation**   - DC-Stimulator Mobile, *NeuroConn* |

*Cognitive basic testing from the Vienna Test System (CogBat)

## 6.4 Stimulation protocol

Transcranial direct current stimulation will be delivered using the device *DC-Stimulator Mobile* from the company *NeuroConn.* The device ensures simple and safe application of the previously set current intensity and current duration. The systems integrated study mode allows effective double-blinding of the investigator and patient. A bifrontal stimulation assembly will be used. The anode is placed on the left dorsolateral prefrontal cortex (*F3* according to the international 10-20 EEG system), the cathode is placed on the right orbital region (*Fp2*). For the treatment arm, a 2 mA current is applied for 20 minutes; for the placebo, a sham stimulation is applied for 20 minutes.

## 6.5 Experimental procedure and time demands on the patients

Following the standard routine diagnostics for inpatients or the first consultation for outpatients (see above), patients will be invited to attend a second consultation. At the beginning of the examination, patients will initially be given a brief explanation about the *DC-Stimulator Mobile*, before application of the device. The device will then be started and the patients asked to complete the “Tower of London”, “N-back verbal”, “Inhib” and “Switch” tests from Schuhfried's Vienna Test System. The selection of test procedures is based on the concept of Miyake's executive functions described above (2000). Since the duration of the tDCS stimulation is shorter than the total time required for the procedures, the stimulation is switched off during the course of the examination. The procedure is finished when test procedures have been completed. The total time required to participate in the study beyond the routine diagnostics is approximately 60 minutes for in-patients. This includes both tDCS stimulation and the processing of neuropsychological procedures. The time required for outpatients is approximately 2 hours for the first consultation and approximately 1 hour for the second consultation, i.e. a total of 3 hours for the entire study period.

## 6.6 Data protection policy

The names of the patients and all other confidential information are subject to medical confidentiality and the provisions of the German Federal Data Protection Act (BDSG which stands for the “Bundesdatenschutzgesetz”). The data is collected, stored and evaluated under a pseudonym. Third parties do not have access to original medical records. The pseudonymisation of the data takes place from the beginning of participation in the study. Each patient is assigned an individual code. The pseudonymisation key is kept on file in the administration centre of the Karlsbad-Langensteinbach Clinic and is not made available to anyone outside of the clinic. The collection, analysis and storage of data will be carried out in the administration centre of the study. No external centres or other bodies will be involved. The data is archived for a period of 10 years.

## 6.7 Intended start and expected completion of the study

The study is scheduled to begin in March 2018. The study is expected to be completed in March 2021.

# 7. Effect/Benefits

There are no direct benefits for patients as a result of participating in the study. Short-term improvements in executive function disorders are anticipated, but are unlikely to persist beyond the duration of the study. The proposed study will, however, also benefit the common good to the extent that it will improve our understanding of executive function disorders in patients with schizophrenia and, in the longer term, lead to improved treatment options for these disorders. Patients, inpatient, day-care or outpatient treatments, are neither advantaged nor disadvantaged by participation in the study.

# 8. Time commitment/risks

Tests administered during the study (questionnaires, neuropsychological tests) are behavioural tests that require a certain degree of mental effort, but are unlikely to pose any risks to the patients.

If the *DC-Stimulator Mobile* is used properly, there is almost no risk to the test person. The following side effects may however be observed:

1. General: itching, tingling, headache, burning sensation of the skin, discomfort, phosphenes (short flashes of light), fatigue, nausea or vomiting, temporary dizziness.

2. In patients with depression the following have been observed in some rare cases: Euphoria, hypomania, nausea, confusion, anxiety, insomnia.

# 9. Study design

The study is a monocentric, prospective, interventional, controlled, randomised, double-blind study.

# 10. Inclusion/exclusion criteria

The inclusion criteria are as follows:

- Patients (age > 18 years) with an F2x diagnosis, i.e. schizophrenia, schizotypal disorder, delusional disorder or schizoaffective disorder in inpatient, day-care or outpatient settings in the Department of Psychiatry and Psychotherapy at the SRH Clinic Karlsbad-Langensteinbach , as well as any persons with the above-mentioned disorders who are not treated in the Department of Psychiatry and Psychotherapy at the SRH Clinic Karlsbad-Langensteinbach but who wish to participate in the study.
- Deficiencies in executive functions (verbal working memory, response inhibition, planning ability, cognitive flexibility).

The exclusion criteria are as follows:

- Impaired intelligence (i.e. IQ ≤ 85)
- Neurological diseases that impact cognition
- Drug use within the last 8 weeks
- Patients that are minors
- Patients of legal age who are unable to give their informed consent

# 11. Criteria for withdrawal

## Individual criteria for withdrawal

All study participants can withdraw from the study at any time and without justification and without prejudice to ongoing medical care by withdrawing their consent to participate in the study. Participation in the study may also be discontinued if this becomes necessary for clinical reasons in the Department of Psychiatry and Psychotherapy at the SRH Clinic Karlsbad-Langensteinbach. Participation will also be discontinued if side effects arise as a result of using the DC device, which precludes the stimulation from being continued.

## 11. 2 Criteria for withdrawal of the entire study

None.

# 12. Randomisation procedure

In order to ensure effective double-blinding, randomisation is carried out in collaboration with the manufacturer of the direct current device, the Neuroconn company. The company compiles a list of codes, half of which correspond to verum stimulations and the other half to sham stimulations. The sequence of the codes is then scrambled using randomly generated numbers. Before starting each stimulation, one of these codes is entered, upon which either a verum or a sham stimulation is started. This means that neither the investigator nor the patient knows what type of stimulation is being applied.

# 13. Statistical design

## 13.1 Proposed size of the patient population

The proposed study, will compare mean values (respective average values from the neuropsychological tests) in a two-factor design across independent groups pre-post (2*2 mixed-model design). The statistical hypothesis is as follows:

Statistical hypothesis: There is an interaction between *group*time* in the dependent variable *Performance in the neuropsychological test procedures.*

The size of the patient population required is therefore estimated as a function of the power of the study (1-β), the level of statistical significance (α) and the magnitude of the effect expected (Cohen's d) (Cohen, 1988). The size of the patient population required for the study was estimated using the *G-Power 3* software (Faul, Erdfelder, Lang & Buchner, 2007). To achieve a power of 1-β = 0.9, with a significance level of α = 0.05 and an expected mean effect of d = 0.5, the study needs to enrol n = 23 patients in each group. Assuming a drop-out rate of approximately 10%, the study needs to enrol n = 25 probands in each group. The study will therefore require:

1. 25 patients in the verum stimulation arm
2. 25 patients in the sham stimulation arm

## 13.2 Statistical analysis

The statistical analysis will be performed on pseudonymised data. Variables of patient characteristics and dependent variables will be analysed using descriptive statistics. For the inferential statistical evaluation of the data, mean value comparisons between the two groups will be performed using pre/post comparisons. Each of the dependent variables will be analysed using on a two-factor mixed-model ANOVA with the between-subject factor group (*verum stimulation* vs. *sham stimulation*) and the within-subject factor time interval (*pre* vs. *post*). Any potential interactions will be further explored by using *a priori* planned comparisons (e.g. t-tests). Where appropriate, several dependent variables will be grouped together to reduce the number of analyses required. If the assumptions for parametric analyses are not satisfied (normal distribution of the error components, homoscedasticity of the error variances, independence of the measured values), the means of individual dependent variables will be compared using a nonparametric test (e.g. Mann-Whitney U-test). If multiple statistical methods are used (t-tests, Mann-Whitney U-tests), the alpha level will be adjusted (Bonferroni correction or Bonferroni Holm procedure) to reflect the overall alpha. The magnitude of individual effects will also be determined (Cohen, 1988) to quantify any potential differences obtained.

# 13. Legal and ethical considerations

The study will be conducted in accordance with the current version of the Helsinki declaration. The study protocol will be submitted for review to the ethics committee of the Heidelberg faculty prior to the start of the study. The names of the patients and all other confidential information are subject to medical confidentiality and the provisions of the State Data Protection Act of Baden-Württemberg (LDSG which stands for Landesdatenschutzgesetzes Baden-Württemberg) or the German Federal Data Protection Act (BDSG which stands for the “Bundesdatenschutzgesetz”). Patient data may only be forwarded in pseudonymised form. Third parties will not be given access to original documents.

Patients' participation is voluntary. Consent may be withdrawn at any time without providing any justification and without prejudice to ongoing medical care. The study participants are informed both verbally and in writing prior to the start of the study about the nature and scope of the planned investigation, in particular about the potential benefits to their health and any risks involved. The patient gives his/her informed consent by signing the declaration of consent. Should the patient withdraw from the study, any previously obtained material (data) will be destroyed. Alternatively, the patient may be asked whether he/she agrees to the use of their data in the analysis.

The proposed study is classed as Other Study according to § 23b of the German Medical Devices Act (MPG which stands for “Medizinproduktegesetz”). The direct current stimulation unit *DC-Stimulator Mobile* and the associated software have been CE certified by the manufacturer NeuroConn. The current CE certification and instructions for use will be submitted with the new application. The proposed study does not aim to collect data on the performance of the medical device (the *DC-Stimulator Mobile* unit in this case). The medical device (the *DC-Stimulator Mobile* unit in this case) will be used within the scope of its intended purpose (the study is not designed to assess conformity, i.e. the medical device will not be further developed into a new CE-marked medical device).No additional invasive or stressful examinations will be performed.

# 14. Funding, immaterial support, institutional affiliations, potential conflicts of interest, incentives for participants, compensation

The study is funded by the SRH Holding Foundation. The funding provided is comparatively low in proportion to the costs of the project. The study has not received any other financial support from third parties. *DC-Stimulator Mobile* units were purchased from NeuroConn through Hasomed in accordance with standard purchase procedures. In order to ensure effective double-blinding, randomisation codes are provided by NeuroConn as a service. There will be no further collaboration with NeuroConn beyond this. There are no conflicts of interest. Patients do not receive an expense allowance for their participation in the study. The business liability insurance (insurer: R + V Allgem. Versicherung AG) also covers the fault-based liability (= legal liability) of SRH Holding Karlsbad-Langensteinbach Clinic GmbH for clinical studies. The overall amounts insured, for individual claims, under the SRH Holding group contract are as follows: € 7,500,000 for personal injury and property damage and € 200,000 for financial losses.

**Literature**

- Aschenbrenner, S., Kaiser, S., Pfüller, U., Roesch-Ely, D. & Weisbrod, M. (2012). *Wiener Testsystem: Testset Kognitive Basistestung (CogBat)*. Mödling: Schuhfried.
- Beblo, T., Kunz, M., Lautenbacher, S., Albert, A. & Aschenbrenner, S. (2011). *Wiener Testsystem: Fragebogen zur geistigen Leistungsfähigkeit*. Mödling: Schuhfried.
- Bikson, M., Grossman, P., Thomas, C., Zannou, A. L., Jiang, J., Adnan, T.et al. (2016). Safety of Transcranial Direct Current Stimulation: Evidence Based Update 2016. *Brain Stimul, 9*(5), 641-661.
- Bodatsch, M. (2014). Transkranielle Gleichstromstimulation bei psychischen Störungen. In J. Kuhn & W. Gaebel (Hrsg.), *Therapeutische Stimulationsverfahren für psychiatrische Erkrankungen* (S. 155-169). Stuttgart: Kohlhammer.
- Brunoni, A. R. & Vanderhasselt, M. A. (2014). Working memory improvement with non-invasive brain stimulation of the dorsolateral prefrontal cortex: a systematic review and meta-analysis. *Brain Cogn, 86*, 1-9.
- Cicerone, K., Levin, H., Malec, J., Stuss, D. & Whyte, J. (2006). Cognitive rehabilitation interventions for executive function: moving from bench to bedside in patients with traumatic brain injury. *J Cogn Neurosci, 18*(7), 1212-1222.
- Cohen, D. (1988). *Statistical Power Analysis for the Behavioral Sciences* (2nd). Hillsdale: Lawrence Erlbaum Associates.
- Dickinson, D., Ramsey, M. E. & Gold, J. M. (2007). Overlooking the obvious: a meta-analytic comparison of digit symbol coding tasks and other cognitive measures in schizophrenia. *Arch Gen Psychiatry, 64*(5), 532-542.
- Dockery, C. A., Hueckel-Weng, R., Birbaumer, N. & Plewnia, C. (2009). Enhancement of planning ability by transcranial direct current stimulation. *J Neurosci, 29*(22), 7271-7277.
- Exner, C. & Lincoln, T. (2012). *Neuropsychologie schizophrener Störungen*. Göttingen: Hogrefe.
- Faul, F., Erdfelder, E., Lang, A. G. & Buchner, A. (2007). G*Power 3: a flexible statistical power analysis program for the social, behavioral, and biomedical sciences. *Behav Res Methods, 39*(2), 175-191.
- Fisher, M., Herman, A., Stephens, D. B. & Vinogradov, S. (2016). Neuroscience-informed computer-assisted cognitive training in schizophrenia. *Ann N Y Acad Sci, 1366*(1), 90-114.
- Fregni, F., Boggio, P. S., Nitsche, M., Bermpohl, F., Antal, A., Feredoes, E.et al. (2005). Anodal transcranial direct current stimulation of prefrontal cortex enhances working memory. *Exp Brain Res, 166*(1), 23-30.
- Glahn, D. C., Laird, A. R., Ellison-Wright, I., Thelen, S. M., Robinson, J. L., Lancaster, J. L.et al. (2008). Meta-analysis of gray matter anomalies in schizophrenia: application of anatomic likelihood estimation and network analysis. *Biol Psychiatry, 64*(9), 774-781.
- Harvey, P. D., Green, M. F., Bowie, C. & Loebel, A. (2006). The dimensions of clinical and cognitive change in schizophrenia: evidence for independence of improvements. *Psychopharmacology (Berl), 187*(3), 356-363.
- Hautzinger, M., Keller, F. & Kühner, C. (2006). *Beck Depressions- Inventar (BDI-II). Revision*. Frankfurt/Main: Harcourt Test Services.
- Hill, A. T., Fitzgerald, P. B. & Hoy, K. E. (2016). Effects of Anodal Transcranial Direct Current Stimulation on Working Memory: A Systematic Review and Meta-Analysis of Findings From Healthy and Neuropsychiatric Populations. *Brain Stimul, 9*(2), 197-208.
- Hoy, K. E., Arnold, S. L., Emonson, M. R., Daskalakis, Z. J. & Fitzgerald, P. B. (2014). An investigation into the effects of tDCS dose on cognitive performance over time in patients with schizophrenia. *Schizophr Res, 155*(1-3), 96-100.
- Kanning, U. P. (2009). *Inventar Sozialer Kompetenzen (ISK)*. Göttingen: Hogrefe.
- Kuhl, J. & Kazén, M. (2009). *Persönlichkeits-Stil-und-Störungs-Inventar (PSSI). Manual.* (Bd. 2nd Ed.). Göttingen: Hogrefe.
- Lehrl, S. (2005). *Mehrfachwahl-Wortschatz-Intelligenztest MWT-B* (Bd. 5. Auflage). Balingen: Spitta Verlag.
- Leucht, S., Vauth, R., Olbrich, H. M. & Jäger, M. (2014). Schizophrenien und andere psychotische Störungen. In M. Berger (Hrsg.), *Psychische Erkrankungen : Klinik und Therapie* (5. ed., S. 301 - 358). München: Elsevier.
- Mervis, J. E., Capizzi, R. J., Boroda, E. & MacDonald, A. W., 3rd. (2017). Transcranial Direct Current Stimulation over the Dorsolateral Prefrontal Cortex in Schizophrenia: A Quantitative Review of Cognitive Outcomes. *Front Hum Neurosci, 11*, 44.
- Mesholam-Gately, R. I., Giuliano, A. J., Goff, K. P., Faraone, S. V. & Seidman, L. J. (2009). Neurocognition in first-episode schizophrenia: a meta-analytic review. *Neuropsychology, 23*(3), 315-336.
- Minzenberg, M. J., Laird, A. R., Thelen, S., Carter, C. S. & Glahn, D. C. (2009). Meta-analysis of 41 functional neuroimaging studies of executive function in schizophrenia. *Arch Gen Psychiatry, 66*(8), 811-822.
- Miyake, A., Friedman, N. P., Emerson, M. J., Witzki, A. H., Howerter, A. & Wager, T. D. (2000). The unity and diversity of executive functions and their contributions to complex "Frontal Lobe" tasks: a latent variable analysis. *Cogn Psychol, 41*(1), 49-100.
- Montgomery, S. A. & Asberg, M. (1979). A new depression scale designed to be sensitive to change. *Br J Psychiatry, 134*, 382-389.
- Müller, S. V. (2013). *Störungen der Exekutivfunktionen*. Göttingen: Hogrefe.
- Müller, S. V., Hildebrandt, H. & Münte, F. (2004). *Kognitive Therapie bei Störungen der Exekutivfunktionen - ein Therapiemanual*. Göttingen: Hogrefe.
- Nienow, T. M., MacDonald, A. W., 3rd & Lim, K. O. (2016). TDCS produces incremental gain when combined with working memory training in patients with schizophrenia: A proof of concept pilot study. *Schizophr Res, 172*(1-3), 218-219.
- Nitsche, M. A. & Paulus, W. (2000). Excitability changes induced in the human motor cortex by weak transcranial direct current stimulation. *J Physiol, 527 Pt 3*, 633-639.
- Nitsche, M. A. & Paulus, W. (2001). Sustained excitability elevations induced by transcranial DC motor cortex stimulation in humans. *Neurology, 57*(10), 1899-1901.
- Nuechterlein, K. H., Subotnik, K. L., Green, M. F., Ventura, J., Asarnow, R. F., Gitlin, M. J.et al. (2011). Neurocognitive predictors of work outcome in recent-onset schizophrenia. *Schizophr Bull, 37 Suppl 2*, S33-40.
- Pope, P. A., Brenton, J. W. & Miall, R. C. (2015). Task-Specific Facilitation of Cognition by Anodal Transcranial Direct Current Stimulation of the Prefrontal Cortex. *Cereb Cortex, 25*(11), 4551-4558.
- Rindermann, H. (2009). *Emotionaler Kompetenz Fragebogen (EKFS)*. Göttingen: Hogrefe.
- Smith, R. C., Boules, S., Mattiuz, S., Youssef, M., Tobe, R. H., Sershen, H.et al. (2015). Effects of transcranial direct current stimulation (tDCS) on cognition, symptoms, and smoking in schizophrenia: A randomized controlled study. *Schizophr Res, 168*(1-2), 260-266.
- Tsang, H. W., Leung, A. Y., Chung, R. C., Bell, M. & Cheung, W. M. (2010). Review on vocational predictors: a systematic review of predictors of vocational outcomes among individuals with schizophrenia: an update since 1998. *Aust N Z J Psychiatry, 44*(6), 495-504.
- Wittchen, H.-U., Zaudig, M. & Fydrich, T. (1997). *Strukturiertes Klinisches Interview für DSM-IV.* Göttingen Hogrefe.
