# Supplementary material for: Acute effects of a single dose of 2 mA of anodal transcranial direct current stimulation over the left dorsolateral prefrontal cortex on executive functions in patients with schizophrenia—A randomized controlled trial
Source: PLoS One. 2021 Jul 16;16(7):e0254695. doi: 10.1371/journal.pone.0254695 (PMC8284793; doi:10.1371/journal.pone.0254695)
Supplement: S3 File — Report to the Ethics Committee German Version. (DOC) [file pone.0254695.s005.doc]

# 1. Deckblatt

Titel des Projektes: Akute Effekte der transkraniellen Gleichstromstimulation auf Störungen der Exekutivfunktionen bei Patienten mit Schizophrenie

Leiter der Studie: Prof. (apl) Dr. med. Matthias Weisbrod

Co-Leiter der AG Neurokognition

Klinik für Allgemeine Psychiatrie

Universitätsklinikum Heidelberg

Chefarzt

Abteilung für Psychiatrie und Psychotherapie

SRH Klinikum Karlsbad-Langensteinbach

Telefon: 07202-613342

Email: [matthias.weisbrod@srh.de](mailto:matthias.weisbrod@srh.de)

Weitere Beteiligte: Dr. phil. Dipl. Psych. Steffen Aschenbrenner

Leitender Psychologe

Abteilung für Klinische Psychologie und Neuropsychologie

SRH Klinikum Karlsbad-Langensteinbach

Telefon: 07202-613602

Email: [steffen.aschenbrenner@srh.de](mailto:steffen.aschenbrenner@srh.de)

Dr. rer. nat. Dipl. Psych. Thomas Schilling

Abteilung für Klinische Psychologie und Neuropsychologie

SRH Klinikum Karlsbad-Langensteinbach

Telefon: 07202-613058

Email: [thomas.schilling@srh.de](mailto:thomas.schilling@srh.de)

Biometriker: An der Studie ist kein Biometriker beteiligt oder im Rahmen einer statistischen Beratung in die Studienplanung eingebunden.

Finanzielle Unterstützung: Die Studie wird durch die SRH Förderstiftung finanziell unterstützt

Datum/Version : 05 Juli 2018/ Version_3

Unterschrift des Studienleiters

Prof. Dr. med. M. Weisbrod

# 2. Zusammenfassung

Kognitive Störungen wie beispielsweise Defizite in den Exekutivfunktionen treten bei Patienten mit Schizophrenie häufig auf und sind wichtigster Prädiktor für die Wiederherstellung bzw. den Erhalt der Arbeitsfähigkeit. Der Therapie dieser Störungen kommt daher eine hohe Bedeutung zu. Eine vielversprechende neue therapeutische Option ist die neuromodulatorische Technik der transkraniellen Gleichstromstimulation. Ziel der zur Begutachtung eingereichten Studie ist es, kurzfristige Effekte der Gleichstromstimulation auf Störungen der Exekutivfunktionen bei Patienten mit Schizophrenie zu untersuchen.

Untersuchungsdesign: Multivariates, zweifaktorielles mixed-modell design mit zweifach gestuftem between-subject Faktor *Gruppe* (Verum-Stimulation vs. Sham-Stimulation) und zweifach gestuftem within-subject Faktor *Zeit* (prä vs. post). Vor und nach der Stimulation bearbeiten die Patienten mehrere neuropsychologische Verfahren zur Erfassung von Exekutivfunktionen.

Stichprobe: Insgesamt 50 Patienten mit Diagnose F2.x (d.h. Schizophrenie, Schizotype Störung, Wahnhafte Störung oder Schizoaffektive Störung) randomisiert aufgeteilt auf zwei Gruppen mit je 25 Patienten. Aufgrund der geplanten Studie wird weder eine medikamentöse Behandlung von Patienten eingeleitet, noch wird eine bestehende medikamentöse Behandlung in irgendeiner Art und Weise durch die geplante Studie verändert (z.B. Veränderung der Dosis). Die zeitliche Gesamtbelastung der Patienten für die Studienteilnahme beträgt für stationäre Patienten ca. 60 Minuten, für ambulante Patienten ca. 3 Stunden

Die Studie wird nach den Bestimmungen der Berufsordnung für Ärzte und in Übereinstimmung mit der Deklaration von Helsinki in der aktuellen Fassung von 2013 durchgeführt. Die Patienten werden vor Beginn der Studie schriftlich und mündlich aufgeklärt und geben schriftlich ihr Einverständnis, welches jederzeit, ohne Angabe von Gründen, zurückgezogen werden kann. Die transkranielle Gleichstromstimulation ist bei sachgemäßer Anwendung risikofrei und nebenwirkungsarm.

**Inhaltsverzeichnis**

[1. Deckblatt 1](#__RefHeading___Toc47961050)

[2. Zusammenfassung 3](#__RefHeading___Toc47961051)

[3. Einleitung/Wissenschaftliche Grundlagen 6](#__RefHeading___Toc47961052)

[3.1. Exekutivfunktionen 6](#__RefHeading___Toc47961053)

[3.2 Kognitive Störungen bei Patienten mit Schizophrenie 7](#__RefHeading___Toc47961054)

[3.3 Transkranielle Gleichstromstimulation 9](#__RefHeading___Toc47961055)

[4. Ziel der Studie 11](#__RefHeading___Toc47961056)

[5. Zielkriterien 11](#__RefHeading___Toc47961057)

[6. Studienbedingte Maßnahmen/Studienablauf 12](#__RefHeading___Toc47961058)

[6.1 Rekrutierung der Stichprobe 12](#__RefHeading___Toc47961059)

[6.2 Aufklärung der Probanden 13](#__RefHeading___Toc47961060)

[6.3 Erhebung der psychometrischen und klinischen Daten 13](#__RefHeading___Toc47961061)

[6.4 Standardisierte und normierte neuropsychologische Testverfahren zur Beurteilung der kognitiven Leistungsfähigkeit: 13](#__RefHeading___Toc47961062)

[6.4 Stimulationsverfahren 15](#__RefHeading___Toc47961063)

[6.5 Versuchsablauf und zeitliche Belastung für die Patienten 15](#__RefHeading___Toc47961064)

[6.6 Datenschutzkonzept 16](#__RefHeading___Toc47961065)

[6.7 Geplanter Studienbeginn und voraussichtliches Ende 16](#__RefHeading___Toc47961066)

[7. Wirkung/Nutzen 16](#__RefHeading___Toc47961067)

[8. Belastungen/Risiken 16](#__RefHeading___Toc47961068)

[9. Studientyp (-design) 17](#__RefHeading___Toc47961069)

[10. Einschluss-/Ausschlusskriterien 17](#__RefHeading___Toc47961070)

[11. Abbruchkriterien 18](#__RefHeading___Toc47961071)

[11.1 Individuelle Abbruchkriterien 18](#__RefHeading___Toc47961072)

[11. 2 Abbruchkriterien für die gesamte Studie 18](#__RefHeading___Toc47961073)

[12. Randomisierungsverfahren 18](#__RefHeading___Toc47961074)

[13. Statistisches Design 19](#__RefHeading___Toc47961075)

[13.1 Stichprobenumfangsplanung 19](#__RefHeading___Toc47961076)

[13.2 Statistische Analyse 19](#__RefHeading___Toc47961077)

[13. Rechtliche und ethische Aspekte 20](#__RefHeading___Toc47961078)

[14. Finanzierung, ideelle Unterstützung, institutionelle Verbindungen, mögliche Interessenskonflikte, Anreize für Versuchspersonen, Entschädigungen 21](#__RefHeading___Toc47961079)

[15. Literaturverzeichnis 22](#__RefHeading___Toc47961080)

# 3. Einleitung/Wissenschaftliche Grundlagen

Die Schizophrenie ist eine heterogen verlaufende psychiatrische Erkrankung, die oftmals mit massiven Einschränkungen in der Fähigkeit zur Teilhabe am gesellschaftlichen Leben und der eigenständigen Lebensführung einhergeht. Neben den klinisch imponierenden Symptomen wie Wahn, Wahrnehmungsveränderungen, Denk- und Antriebsstörungen leiden die Erkrankten insbesondere unter kognitiven Beeinträchtigungen wie Defiziten in den Exekutivfunktionen. Diese sind langfristig die besten Prädiktoren für die Aufrechterhaltung bzw. die Wiedererlangung von Alltagsfunktionalität und ihrer Rehabilitation kommt somit eine hohe Bedeutung zu. Moderne neurowissenschaftliche Stimulationsverfahren wie die transkranielle Gleichstromstimulation könnten hierzu einen wichtigen Beitrag leisten. Im Rahmen der zur Begutachtung vorgelegten Studie sollen die kurzfristigen Effekte dieser Methode auf Störungen der Exekutivfunktionen bei Patienten mit Schizophrenie untersucht werden. Im Folgenden werden die relevanten theoretischen Hintergründe dargestellt.

## 3.1. Exekutivfunktionen

Unter dem Begriff der Exekutivfunktionen werden in der Neuropsychologie kognitive Steuerungs- und Leitungsfunktionen verstanden. Nach Müller (2013) sind sie „Regulations- und Kontrollmechanismen, die zielorientiertes und situationsangepasstes Verhalten ermöglichen“ sowie metakognitive Prozesse, die der Erreichung eines definierten Zieles oder der Zielerarbeitung dienen. Kennzeichnend für sie sind sowohl Flexibilität im Sinne einer Anpassung an wechselnde Anforderungen als auch Persistenz, d.h. das Verfolgen eines Zieles und die Abschirmung einer Handlung gegenüber Ablenkungen (ebs). Exekutivfunktionen ermöglichen einem Organismus ein zielorientiertes und flexibles Handeln und zwar insbesondere dann, wenn Routinen zur Lösung eines Problems nicht mehr ausreichen.

Den meisten Definitionen von Exekutivfunktionen ist gemein, dass hierunter mehrere kognitive Prozesse subsummiert werden. So unterscheiden beispielsweise Miyake und Kollegen (2000) drei Basismechanismen: 1). Shifting (Wechsel des Aufmerksamkeitsfokus), 2). Updating (das Aktualisieren von Arbeitsgedächtnisinhalten) und 3). Inhibition (die Unterdrückung dominanter Antworttendenzen). Alle drei Basismechanismen sind gemäß des Modelles gemeinsam für die Generierung einer komplexen Exekutivfunktion notwendig. Müller und Kollegen (2004) unterscheiden in einer klinischen Definition der Exekutivfunktionen die Bereiche 1) Arbeitsgedächtnis und Monitoring, 2). Kognitive Flexibilität sowie 3). Problemlösen. Passend zu diesen Definitionen werden Exekutivfunktionen im klinischen Alltag meisten mit Aufgaben zum Arbeitsgedächtnis, zur Flexibilität und zum Wechsel, zur Reaktionsinhibition sowie zur Planung operationalisiert und gemessen.

Neuroanatomisch werden Exekutivfunktionen insbesondere mit dem präfrontalen Kortex in Verbindung gebracht. Eine Vielzahl von klinischen Fallberichten und Untersuchungen zeigt, dass Schädigung in diesem Bereich mit Defiziten in der Planung, dem Arbeitsgedächtnis, der Reaktionsinhibition sowie der Flexibilität und Persistenz einhergehen (Müller, 2013). Gemäß Cicerone und Kollegen (2006) lassen sich die unterschiedlichen Exekutivfunktionen verschiedenen neuroanatomischen Regionen zuordnen. Kognitive Funktionen wie Planung, Überwachung, Wechsel, Arbeitsgedächtnis und Inhibition werden demnach durch den dorsolateralen präfrontalen Kortex (DLPFC) vermittelt. Die Selbstregulationsfähigkeit eines Organismus ist eng mit dem ventromedialen präfrontalen Kortex verknüpft, Antrieb und Aktivierung mit dem mediofrontalen Kortex und metakognitive Prozesse mit den frontalen Polen.

Störungen der Exekutivfunktionen treten bei einer Vielzahl neurologischer und psychiatrischer Erkrankungen auf. Eine Patientengruppe, die häufig unter Defiziten der Exekutivfunktionen leidet, sind Patienten mit Schizophrenie.

## 3.2 Kognitive Störungen bei Patienten mit Schizophrenie

Die Schizophrenie ist eine heterogen verlaufende psychiatrische Erkrankung mit einer weltweiten Prävalenz von ca. 1 %. Klinisch kennzeichnend sind Positiv- (Wahn, Paranoia und Halluzinationen) und Negativsymptome (Verflachung des Affekts, Apathie, Willenslosigkeit, gedankliche Verarmung, Katatonie) sowie desorganisiertes Verhalten (unpassender Affekt, Zerfahrenheit, Verwirrtheit) (Fisher, Herman, Stephens & Vinogradov, 2016; Leucht, Vauth, Olbrich & Jäger, 2014).

Neben diesen Hauptsymptomen wurden bereits vor mehr als hundert Jahren kognitive Störungen bei Patienten mit Schizophrenie beschrieben. So sprach Kraepelin von der *Dementia praecox* und sah einen dementiellen Abbau bereits im jungen Erwachsenenalter mit vielfältigen kognitiven Beeinträchtigungen als eines der kennzeichnenden Symptome dieser Erkrankung an. Epidemiologischen Studien zufolge kommt es bei bis zu 80% aller Erkrankten zu kognitiven Störungen (Leucht et al., 2014). Diese zeigen sich sowohl in der Verminderung des generellen Intelligenzniveaus („IQ“) als auch in der Beeinträchtigung einer Vielzahl kognitiver Teilleistungen: Betroffen sind vor allem die Aufmerksamkeit, das Gedächtnis sowie die Exekutivfunktionen (für eine Übersicht siehe Exner & Lincoln, 2012). Bei den Gedächtnisstörungen imponieren Beeinträchtigungen in der verbalen Lern- und Merkfähigkeit. In der Aufmerksamkeit finden sich Störungen der Verarbeitungsgeschwindigkeit, der Vigilanz sowie der Aufmerksamkeitsselektivität. In den Exekutivfunktionen kommt es zu Defiziten im verbalen Arbeitsgedächtnis, dem Problemlösen sowie der kognitiven Flexibilität (z.B. Dickinson, Ramsey & Gold, 2007; Mesholam-Gately, Giuliano, Goff, Faraone & Seidman, 2009).

Die Defizite in den Exekutivfunktionen legen eine Beteiligung präfrontaler Strukturen an der Pathophysiologie der kognitiven Störung bei Patienten mit Schizophrenie nahe. Studien mit bildgebenden Verfahren konnten dies bestätigen. Patienten mit Schizophrenie weisen im Vergleich zu gesunden Kontrollprobanden ein vermindertes Gehirnvolumen auf, u.a. im dorsolateralen präfrontalen Kortex und auch im medialen Temporallappen sowie in weiteren Hirnregionen (Glahn et al., 2008). In Untersuchungen mittels funktioneller Bildgebung (PET, fMRT) konnte zudem ein verminderter Metabolismus im Bereich des DLPFC nachgewiesen werden. Dieser zeigt sich sowohl im Ruhezustand als auch bei der Bearbeitung von Aufgaben, die Anforderungen an die Exekutivfunktionen stellen und ist in der linken Hemisphäre besonders ausgeprägt (Minzenberg, Laird, Thelen, Carter & Glahn, 2009).

Während in der akuten Phase der Erkrankung die Positivsymptomatik sowie das desorganisierte Verhalten im Vordergrund stehen, sind in der subakuten und chronischen Phase die Negativsymptome sowie die kognitiven Störungen von größerer Bedeutung. Die kognitiven Störungen sind hierbei vom Verlauf der Akutsymptomatik weitgehend unabhängig und zeitlich stabil (Harvey, Green, Bowie & Loebel, 2006). Für eine langfristig erfolgreiche berufliche Rehabilitation und Wiedereingliederung sind kognitive Störungen besonders relevant und der prognostisch wichtigste Prädiktor (Nuechterlein et al., 2011; Tsang, Leung, Chung, Bell & Cheung, 2010). Insbesondere die Exekutivfunktionen sowie die verbale Lern- und Merkfähigkeit sind hierbei von hoher Wichtigkeit (Leucht et al., 2014). In der modernen Therapie von Patienten mit Schizophrenie ist es daher inzwischen Standard, nach erfolgter klinischer Stabilisierung den Behandlungsschwerpunkt auf die Rehabilitation der kognitiven Störungen zu legen. Eine neue therapeutische Strategie mit erheblichem klinischem Potential ist die Anwendung einer neuromodulatorischen Technik wie der transkraniellen Gleichstromstimulation (Englisch: transcraniel Direct Current Stimulation = tDCS).

## 3.3 Transkranielle Gleichstromstimulation

Die transkranielle Gleichstromstimulation gehört zu den Verfahren der interventionellen Neurophysiologie. Zur Anwendung werden i.d.R. zwei Elektroden auf die Kopfhaut des Probanden angebracht und dann ein schwacher Gleichstrom von der oberflächenpositiven Anode zur oberflächennegativen Kathode geleitet. Ziel ist die Modulation neuronaler Aktivität in den anvisierten Hirnregionen im Sinne einer Erregung unter der Anode sowie einer Hemmung unter der Kathode (Bodatsch, 2014).

Bei der tDCS werden kurzfristige von langfristigen Effekten unterschieden. Kurzfristige Effekte scheinen über eine Modulation der spontanen neuronalen Netzwerkaktivität während der Stimulation vermittelt zu sein („Online Effekte“), wobei es zu einer polaritätsabhängigen Verschiebung des Ruhemembranpotentials kommt. Unter der Anode kommt es zu einer Depolarisierung der Neurone, wohingegen unter der Kathode der gegenteilige Effekt auftritt (Nitsche & Paulus, 2000, 2001). Längerfristige, über die eigentliche Stimulationsdauer hinausgehende Wirkungen („Offline Effekte“) beruhen wahrscheinlich auf Mechanismen, die der Langzeitpotenzierung bzw. Langzeitdepression ähneln, und sind glutamaterg vermittelt. Veränderungen in der neuronalen Erregbarkeit zeigen sich bis zu 60 Minuten nach Ende der Stimulation (für eine Übersicht siehe Bodatsch, 2014)

Nach bisherigem Kenntnisstand handelt es sich bei der tDCS um eine sichere, gut verträgliche und nebenwirkungsarme neuromodulatorische Technik (Bikson et al., 2016). Potentielle, vorübergehende Nebenwirkungen sind Juckreiz, Kribbeln, Kopfschmerzen, Hautbrennen, Unbehagen, Phosphene (kurze Lichtblitze), Müdigkeit, Übelkeit, Erbrechen oder kurzzeitiges Schwindelgefühl. Zu Forschungszwecken bieten moderne tDCS Geräte die Möglichkeit neben einer echten Verum-Stimulation ebenfalls eine doppelt verblindete Placebo-Stimulation durchzuführen. Hierdurch können unspezifische Wirkungen, z.B. durch die Zuwendung durch den Behandler, von spezifischen Effekten durch die Stimulation getrennt werden.

### 3.3.1 tDCS und Kognition bei Gesunden

Die Wirkung der transkraniellen Gleichstromstimulation auf kognitive Prozesse bei gesunden Probanden wurde vielfach untersucht. Ein spezieller Fokus nimmt hier das verbale Arbeitsgedächtnis ein, wobei aufgrund neuroanatomischer Überlegen i.d.R. eine anodale Stimulation über dem linken DLPFC erfolgt. Die Ergebnisse sind heterogen, jedoch generell positiv im Sinne einer Verbesserung der Arbeitsgedächtnisleistung (für eine Übersicht siehe Brunoni & Vanderhasselt, 2014; Hill, Fitzgerald & Hoy, 2016). So konnten z.B. Fregni und Kollegen (2005) zeigen, dass anodale tDCS über dem linken DLPFC die Leistung in einer N-Back Arbeitsgedächtnisaufgabe verbessert. Pope et al. (2015) fanden in gleicher Stimulationskonfiguration eine verbesserte Leistung in einer schwierigen jedoch nicht in einer einfachen Arbeitsgedächtnisaufgabe. Neben der Arbeitsgedächtnisleistung wurde ebenfalls der Effekt von tDCS auf weitere Exekutivfunktionen bei Gesunden untersucht. So wurden beispielsweise differentielle, förderliche Effekte von anodaler und kathodaler tDCS über dem linken DLPFC auf die Leistung in der Planungsaufgabe „Tower of London“ gefunden (Dockery, Hueckel-Weng, Birbaumer & Plewnia, 2009).

### 3.3.2 tDCS und Kognition bei Patienten mit Schizophrenie

Die Anwendung der transkraniellen Gleichstromstimulation zur Behandlung kognitiver Defizite bei Patienten mit Schizophrenie ist bislang nur wenig untersucht worden. Aufgrund der häufig beschriebenen Hypofrontalität ist eine potentielle Behandlungsstrategie die anodale Stimulation über dem linken DLPFC (für eine Übersicht siehe z.B. Mervis, Capizzi, Boroda & MacDonald, 2017).

In einer ersten Studie mit einmaliger Stimulation zeigten Hoy und Kollegen (2014), dass eine Dosis von 2 Milliampere (mA) für 20 Minuten appliziert über dem linken DLPFC die Leistung in einer verbalen Arbeitsgedächtnisaufgabe verbesserte. Weitere Untersuchungen mit wiederholter Stimulation konnten die positive Wirkung bestätigen. So fanden Smith und Kollegen (2015) nach fünfmaliger Stimulation des linken DLPFC - ebenfalls mit 2 mA für 20 Minuten - eine Verbesserung des verbalen Arbeitsgedächtnisses sowie der attentionalen Funktionen, jedoch keine Effekte auf das schlussfolgernde Denken/Problemlösen oder die Lernfähigkeit. Nienow und Kollegen (2016) zeigten in einer Pilotstudie, dass die simultane Applikation von tDCS während eines Arbeitsgedächtnistrainings den Trainingseffekt verbessern kann.

Zusammengefasst zeigen die oben zitierten Studien, dass die tDCS kognitive Funktionen bei Gesunden und Patienten mit Schizophrenie verbessern kann. Die bisherigen Studien belegen positive Effekte auf das verbale Arbeitsgedächtnis, wohingegen bislang keine Wirkung auf weitere Bereiche der Exekutivfunktionen wie der Planungsfähigkeit oder der Reaktionsinhibition gezeigt werden konnten. Dies verdeutlicht, dass weitere Forschung nötig ist, um bisherige Stimulationsprotokolle zu verbessern und ggfls. differentielle Therapieindikationen zu entwickeln.

# 4. Ziel der Studie

Übergeordnete Fragestellung des vorgestellten Forschungsvorhabens ist, ob und inwieweit Defizite in den Exekutivfunktionen bei Patienten mit Schizophrenie mittels transkranieller Gleichstromstimulation gebessert werden können. Speziell soll untersucht werden, ob eine einmalige Applikation von tDCS zu kurzfristigen, positiven Effekten auf Störungen der Exekutivfunktionen bei dieser Patientengruppe führt.

Hypothese: Die einmalige Anwendung einer anodalen transkraniellen Gleichstromstimulation mit einer Stromstärke von 2 mA über einen Zeitraum von 20 Minuten über dem linken präfrontalen Kortex führt zu einer kurzfristigen Verbesserung von Störungen von Exekutivfunktionen bei Patienten mit Schizophrenie.

# 5. Zielkriterien

Hauptzielkriterium für die oben genannten Hypothesen ist die Verbesserung der kognitiven Leistungsfähigkeit im Bereich der Exekutivfunktionen bei Patienten mit Schizophrenie, gemessen mit mehreren standardisierten neuropsychologischen Testverfahren. Darüber hinaus existieren keine Nebenzielkriterien.

# 6. Studienbedingte Maßnahmen/Studienablauf

## 6.1 Rekrutierung der Stichprobe

Die Studie wird in der Abteilung Psychiatrie und Psychotherapie des Klinikums Karlsbad-Langensteinbach durchgeführt. Die Rekrutierung der Patientenstichprobe erfolgt über die Stationen. Im Verlauf der routinemäßigen Aufnahmediagnostik werden die Patienten befragt, ob sie an der Studie teilnehmen möchten. In diesem Erstgespräch erfolgt eine Überprüfung der Ein-/Ausschlusskriterien sowie die Aufklärung über die Studie. Sollte der Patient einverstanden sein, erfolgt daraufhin die Terminvereinbarung für die eigentliche Untersuchung.

Zusätzlich zu den stationär behandelten Patienten, werden auch ambulante Patienten eingeschlossen. Interessierte werden zunächst zu einem Erstgespräch eingeladen, in dem die Überprüfung der Ein-/Ausschlusskriterien sowie die Aufklärung über die Studie erfolgt. Wenn der Patient an der Studie teilnehmen möchte, wird er im Anschluss an das Gespräch einige psychologische Testverfahren bearbeiten und mehrere Fragebögen ausfüllen. Hierbei handelt es sich um die Verfahren, welche auch die stationären Patienten im Rahmen der klinischen Routinediagnostik durchlaufen. Daraufhin erfolgt die Terminvereinbarung für die eigentliche Untersuchung unter Stimulation mit dem tDCS-Gerät.

Aufgrund der geplanten Studie wird weder eine medikamentöse Behandlung von Patienten eingeleitet, noch wird eine bestehende medikamentöse Behandlung in irgendeiner Art und Weise durch die geplante Studie verändert (z.B. Veränderung der Dosis). Die Studienteilnahme erfordert weder eine Umstellung der Medikation noch wird eine klinisch notwendige Umstellung hierdurch behindert werden.

## 6.2 Aufklärung der Probanden

Die Patienten werden vor Studienbeginn schriftlich und mündlich über Wesen und Tragweite der geplanten Untersuchung, insbesondere über den möglichen Nutzen für ihre Gesundheit und eventuelle Risiken, aufgeklärt. Ihre Zustimmung wird durch Unterschrift auf der Einwilligungserklärung dokumentiert. Bei Rücktritt von der Studie wird das bereits gewonnene Material vernichtet, oder beim Patient angefragt, ob er mit der Auswertung des Materials einverstanden ist. Die Teilnahme der Patienten an der Untersuchung ist freiwillig; die Zustimmung kann jederzeit, ohne Angabe von Gründen und ohne Nachteile für die weitere medizinische Versorgung, zurückgezogen werden.

## 6.3 Erhebung der psychometrischen und klinischen Daten

Zur Verifizierung der Diagnose wird ein strukturiertes Interview (SKID-I und –II bzw. Kurzformen hiervon) durchgeführt. Darüber hinaus werden Fragebögen und verschiedene neuropsychologische Testverfahren bearbeitet. Die Untersuchung umfasst folgende Verfahren:

## 6.4 Standardisierte und normierte neuropsychologische Testverfahren zur Beurteilung der kognitiven Leistungsfähigkeit:

Alle Patienten werden im Rahmen der klinischen Routinediagnostik, oder während eines zusätzlichen ambulanten Termins ,vor Beginn der Untersuchung das Testset COGBAT® (Aschenbrenner, Kaiser, Pfüller, Roesch-Ely & Weisbrod, 2012) bearbeiten. Dieses enthält eine Zusammenstellung wichtiger neuropsychologischer Dimensionen zur Abklärung des kognitiven Status von Patienten mit psychischen Erkrankungen. Es beinhaltet die Dimensionen Aufmerksamkeit, Gedächtnis, Exekutivfunktionen und Verarbeitungsgeschwindigkeit. Ein besonderer Fokus wird auf den Leistungen in den Untertests zur Planungsfähigkeit („Tower of London“), zum verbalen Arbeitsgedächtnis („Nback-Verbal“) sowie der Reaktionsinhibition („INHIB“) liegen. Zusätzlich wird der Test „SWITCH“ zur Erfassung der kognitiven Flexibilität aus dem Wiener Testsystem zum Einsatz kommen. Für eine Übersicht der verwendeten Verfahren siehe Tabelle 1.

Tabelle 1: Übersicht diagnostischer, therapeutischer und interventioneller Verfahren

| **Klinische Diagnostik und Psychopathologie**   - Soziodemografisches Interview - SKID-I (Wittchen, Zaudig & Fydrich, 1997) - SKID-II - Montgomery-Asberg Depression Rating Scale (MADRS, Montgomery & Asberg, 1979) - Beck Depressions Inventar (BDI-II, Hautzinger, Keller & Kühner, 2006) - Persönlichkeitsstil- und Störungsinventar (PSSI, Kuhl & Kazén, 2009) - Inventar Sozialer Kompetenz (ISK, Kanning, 2009) - Emotionaler Kompetenzfragebogen (EKFS, Rindermann, 2009) - Fragebogen zur kognitiven Leistungsfähigkeit* (FLei, Beblo, Kunz, Lautenbacher, Albert & Aschenbrenner, 2011) |
| --- |
| **Prämorbide Intelligenz**   - MWT-B (Lehrl, 2005) |
| **Neuropsychologische diagnostische Verfahren**  **Wiener Testsystem**  *Informationsverarbeitungsgeschwindigkeit*   - Trail Making Test Langensteinbacher Version*   *Aufmerksamkeit*   - Geteilte Aufmerksamkeit-WAFG* - Alertness-WAFA*   *Gedächtnis*   - FGT*   *Exekutivfunktionen*   - Arbeitsgedächtnis-Nback verbal* - Inhibition-INHIB Go-Nogo* - Kognitive Flexibilität-Trail Making Test Langensteinbacher Version* - Kognitive Flexibilität- SWITCH - Konvergentes Planen-Tower of London-Freiburger Version* |
| **Neurophysiologische Stimulation**   - DC-Stimulator Mobile, *NeuroConn* |

*Kognitive Basistestung aus dem Wiener Testsystem (CogBat

## 6.4 Stimulationsverfahren

Für die transkranielle Gleichstromstimulation wird das Gerät *DC-Stimulator Mobile* der Firma *NeuroConn* verwendet. Dieses ermöglicht eine einfache und sichere Applikation der vorher eingestellten Stromstärke und Stromdauer. Aufgrund des integrierten Studienmodus ist eine effektive doppelte Verblindung von Versuchsleiter und Patient möglich. Es wird eine bifrontale Stimulationsmontage verwendet. Die Anode wird auf dem linken dorsolateralen präfrontalen Kortex platziert (*F3* gemäß internationalem 10-20 EEG System), die Kathode rechtsorbital *(Fp2*). In der aktiven Bedingung wird für 20 Minuten 2 mA Strom appliziert, in der Placebo Bedingung erfolgt eine Scheinstimulation für eine Dauer von 20 Minuten.

## 6.5 Versuchsablauf und zeitliche Belastung für die Patienten

Im Anschluss an die standardmäßige Routinediagnostik bei den stationären Patienten bzw. des ersten Termins bei den ambulanten Patienten (s.o.) werden die Patienten zu einem zweiten Termin eingeladen. Zu Beginn der Untersuchung wird ihnen der *DC-Stimulator Mobile* erklärt und angelegt. Anschließend wird das Gerät gestartet und die Patienten werden gebeten, die Verfahren „Tower of London“, „Nback-Verbal“, „Inhib“ und „Switch“ aus dem Wiener Testsystem von Schuhfried zu bearbeiten. Die Auswahl der Testverfahren orientiert sich an dem oben beschriebenen Konzept der Exekutivfunktionen von Miyake (2000). Da die Dauer der tDCS-Stimulation kürzer ist als die Gesamtbearbeitungszeit der Verfahren, wird die Stimulation im Laufe der Untersuchung abgeschaltet. Nach Abschluss der Testverfahren ist der Versuch beendet. Der gesamte über die Routinediagnostik hinausgehende Zeitaufwand für die Teilnahme an der Studie beträgt für die stationären Patienten ca. 60 Minuten. Dies umfasst sowohl die tDCS-Stimulation als auch die Bearbeitung der neuropsychologischen Verfahren. Der zeitliche Aufwand für die ambulanten Patienten beträgt ca. 2 Stunden für den ersten Termin und ca. 1 Stunde für den zweiten Termin, d.h. insgesamt 3 Stunden für die gesamte Studienteilnahme.

## 6.6 Datenschutzkonzept

Die Namen der Patienten und alle anderen vertraulichen Informationen unterliegen der ärztlichen Schweigepflicht und den Bestimmungen des Bundesdatenschutzgesetzes (BDSG). Die Daten werden pseudonymisert erhoben, gespeichert und ausgewertet. Dritte erhalten kei­nen Einblick in Originalkrankenunterlagen. Die Pseudonymisierung der Daten erfolgt ab Beginn der Studienteilnahme. Dem Patienten wird ein individueller Code zugeordnet. Der Pseudonymisierungsschlüssel wird in der Studienverwaltung im Klinikum Karlsbad-Langensteinbach aufbewahrt und Niemanden außerhalb zugänglich gemacht. Die Erhebung, Auswertung und Aufbewahrung der Daten erfolgt in der Studienverwaltung. Es sind keine externen Zentren oder weitere Stellen beteiligt. Die Daten werden für einen Zeitraum von 10 Jahren aufbewahrt.

## 6.7 Geplanter Studienbeginn und voraussichtliches Ende

Geplanter Studienbeginn ist März 2018. Voraussichtliches Ende der Studie ist März 2021.

# 7. Wirkung/Nutzen

Für die Patienten ergibt sich durch die Studienteilnahme kein direkter Nutzen. Zu erwarten sind kurzfristige Verbesserungen von Störungen der Exekutivfunktionen, die jedoch voraussichtlich nicht über die Studiendauer hinaus anhalten werden. Durch die geplante Studie hinaus ergibt sich jedoch dergestalt ein Gemeinnutzen, dass hierdurch das Verständnis für Störungen der Exekutivfunktionen bei Patienten mit Schizophrenie gebessert werden kann und längerfristig verbesserte Behandlungsoptionen für diese Störungen entwickelt werden können. Den Patienten entstehen durch die Teilnahme weder Vor- noch Nachteile für die teil-/stationäre bzw. ambulante Behandlung.

# 8. Belastungen/Risiken

Die Messungen (Fragebögen, neuropsychologische Testverfahren) erfolgen behavioral und erfordern eine gewisse geistige Beanspruchung, es sind jedoch keinerlei Risiken für den Patienten zu erwarten.

Bei sachgemäßer Anwendung des *DC-Stimulator Mobile* ist eine Gefährdung des Probanden durch das Gerät nahezu ausgeschlossen. Die folgenden Nebenwirkungen können auftreten:

1. Allgemein: Juckreiz, Kribbeln, Kopfschmerz, Hautbrennen, Unbehagen, Phosphene (kurze Lichtblitze), Müdigkeit, Übelkeit oder Erbrechen, kurzzeitiges Schwindelgefühl

2. Bei Patienten mit Depression ist in seltenen Fällen aufgetreten: Euphorie, Hypomanie, Übelkeit, Verwirrtheit, Angst, Schlaflosigkeit.

# 9. Studientyp (-design)

Es handelt sich um eine monozentrische, prospektive, interventionelle, kontrollierte, randomisierte, doppelverblindete Studie.

# 10. Einschluss-/Ausschlusskriterien

Es gelten die folgenden Einschlusskriterien:

- PatientInnen (Alter > 18 Jahre) mit Diagnose F2x, d.h. Schizophrenie, Schizotype Störung, Wahnhafte Störung oder Schizoaffektive Störung in stationärer, teilstationärer oder ambulanter Behandlung in der Abteilung für Psychiatrie und Psychotherapie des SRH Klinikums Karlsbad-Langensteinbach sowie Personen mit den oben genannten Störungen, die nicht in der der Abteilung für Psychiatrie und Psychotherapie des SRH Klinikums Karlsbad-Langensteinbach behandelt werden und dennoch an der Studie teilnehmen möchten.
- Defizite in den Exekutivfunktionen (verbales Arbeitsgedächtnis, Reaktionsinhibition, Planungsfähigkeit, kognitive Flexibilität).

Es gelten die folgenden Ausschlusskriterien:

- Intelligenzminderung (d.h. IQ ≤ 85)
- Neurologische Erkrankungen, die die Kognition beeinflussen
- Drogenkonsum innerhalb der letzten 8 Wochen.
- Minderjährige Patienten
- Einwilligungsunfähige volljährige Patienten

# 11. Abbruchkriterien

## Individuelle Abbruchkriterien

Alle Studienteilnehmer können zu jedem Zeitpunkt und ohne Nennung von Gründen die Studienteilnahme abbrechen, in dem sie ihre Einwilligung zur Studienteilnahme widerrufen, ohne dass ihnen für die weitere medizinische Versorgung ein Nachteil entsteht. Die Studienteilnahme wird zudem abgebrochen, wenn dies aus klinischen Erwägungen in der Abteilung für Psychiatrie und Psychotherapie des SRH Klinikums Karlsbad-Langensteinbach notwendig ist. Auch wird die Teilnahme abgebrochen, wenn es unter der Anwendung mit dem Gleichstromgerät zu Nebenwirkungen kommt, die ein Weiterführen der Stimulation ausschließen.

## 11. 2 Abbruchkriterien für die gesamte Studie

Keine.

# 12. Randomisierungsverfahren

Um eine effektive doppelte Verblindung zu gewährleisten, erfolgt die Randomisierung in Kooperation mit dem Hersteller des Gleichstromgerätes, der Firma Neuroconn. Dieser erstellt eine Liste von Codes, die zur Hälfte eine Verum- und zur anderen Hälfte eine Sham-Stimulation kodieren. Die Codes werden dann mittels zufällig generierter Zahlen in ihrer Reihenfolge durchmischt. Vor Beginn jeder Stimulation wird einer der Codes eingegeben, woraufhin entweder eine Verum- oder eine Sham-Stimulation startet. Hierdurch ist weder dem Versuchsleiter noch dem Patienten bekannt, welche Art von Stimulation erfolgt.

# 13. Statistisches Design

## 13.1 Stichprobenumfangsplanung

In der vorliegenden Studie werden Mittelwerte (jeweilige gemittelte Werte aus den neuropsychologischen Tests) in einem zweifaktoriellen Design zwischen unabhängigen Gruppen prä-post verglichen (2*2 mixed-modell design). Die statistische Hypothese ist wie folgt:

Statistische Hypothese: Es findet sich eine Interaktion *Gruppe***Zeit* in der abhängigen Variable *Leistung in den neuropsychologischen Testverfahren*.

Die resultierende Stichprobengröße wird in Abhängigkeit von der gewünschten Power (1-β), dem Signifikanzniveau (α) und der Effektgröße (Cohen’s d) berechnet (Cohen, 1988). Zur Berechnung der Stichprobengröße wurde das Programm *G-Power 3* herangezogen (Faul, Erdfelder, Lang & Buchner, 2007). Bei einer gewünschten Power von 1-β = 0,9, einem Signifikanzniveau von α = 0,05 und einem erwarteten mittleren Effekt von d = 0,5 errechnet sich eine benötigte Stichprobengröße von n = 23 pro Gruppe. Unter Berücksichtigung einer Drop-out Rate von ca. 10% berechnet sich ein Stichprobenumfang von n = 25 Probanden pro Gruppe. Somit ergibt sich die folgende Stichprobenumfangsberechnung:

1. 25 Patienten in der Bedingung Verum-Stimulation
2. 25 Patienten in der Bedingung Sham-Stimulation

## 13.2 Statistische Analyse

Die statistische Analyse wird pseudonymisiert durchgeführt. Personencharakterisierende Variablen sowie abhängige Variablen werden mithilfe deskriptiver Statistik dargestellt. Zur inferenzstatistischen Auswertung der Daten werden Mittelwertsvergleiche zwischen den beiden Gruppen im prä/post Vergleich durchgeführt. Hierfür wird eine zweifaktorielle mixed-modell ANOVA mit dem between-subject Faktor Gruppe (*Verum-Stimulation* vs. *Sham-Stimulation*) und dem within-subject Faktor Zeit (*prä* vs. *post*) für die einzelnen abhängigen Variablen durchgeführt. Interaktionen werden mittels *a priori* geplanter Kontraste (z.B. t-tests) weitergehend aufgelöst. Wo dies sinnvoll ist, wird über mehrere abhängige Variablen aggregiert, um die Anzahl an notwendigen Analysen zu reduzieren. Sind die Voraussetzungen parametrischer Verfahren nicht erfüllt (Normalverteilung der Fehlerkomponenten, Homoskedastizität der Fehlervarianzen, Unabhängigkeit der Messwerte), wird getrennt für jede abhängige Variable auf nonparametrische Mittelwertsvergleiche (z.B. Mann-Whitney U-test) zurückgegriffen. Im Falle der mehrfachen Anwendung statistischer Verfahren (t-tests, Mann-Whitney U-tests) wird das Alpha Niveau adjustiert (Bonferroni Korrektur bzw. Bonferroni-Holm-Prozedur), um einer Alpha Akkumulierung Rechnung zu tragen. Zusätzlich werden Effektgrößen berechnet (Cohen, 1988) um die Quantität der Unterschiede zu bestimmen.

# 13. Rechtliche und ethische Aspekte

Die Untersuchung wird in Übereinstimmung mit der Deklaration von Helsinki in ihrer aktuellen Fassung durchgeführt. Das Studienprotokoll wird vor Studienbeginn der Ethikkommission der Medizinischen Fakultät Heidelberg zur Begutachtung vorgelegt. Die Namen der Patienten und alle anderen vertraulichen Informationen unterliegen der ärztlichen Schweigepflicht und den Bestimmungen des Landesdatenschutzgesetzes Baden-Württemberg bzw. des Bundesdatenschutzgesetzes (LDSG BW bzw. BDSG). Eine Weitergabe von Patientendaten erfolgt ggf. nur in pseudonymisierter Form. Dritte erhalten keinen Einblick in Originalunterlagen.

Die Teilnahme der Patienten ist freiwillig. Die Zustimmung kann vom Patienten jederzeit, ohne Angaben von Gründen und ohne Nachteile für die weitere medizinische Versorgung zurückgezogen werden. Die Studienteilnehmer werden vor Studienbeginn schriftlich und mündlich über Wesen und Tragweite der geplanten Untersuchung, insbesondere über den möglichen Nutzen für ihre Gesundheit und eventuelle Risiken, aufgeklärt. Ihre Zustimmung wird durch Unterschrift auf der Einwilligungserklärung dokumentiert. Bei Rücktritt von der Studie wird bereits gewonnenes (Daten-) Material vernichtet oder beim Patient angefragt, ob er/sie mit der Auswertung des Materials einverstanden ist.

Bei der geplanten Studie handelt es sich um eine Sonstige Studie nach § 23b MPG. Das Gleichstromstimulationsgerät DCS Mobile sowie die dazugehöre Software sind durch den Hersteller NeuroConn CE zertifiziert worden. Die aktuelle CE Zertifizierung sowie eine Gebrauchsanweisung werden mit der Neueinreichung mit eingereicht. Die aktuelle Studie verfolgt nicht das Ziel, Daten zur Eignung des Medizinproduktes (hier DCS Mobile) zu erheben. Das Medizinprodukt (hier DCS Mobile) wird in der Studie im Rahmen seiner Zweckbestimmung verwendet (die Studie ist nicht auf eine Konformitätsbewertung ausgerichtet, d.h. das Medizinprodukt wird nicht zu einem neuen CE gekennzeichneten Medizinprodukt weiterentwickelt). Es werden keine zusätzlichen invasiven oder belastenden Untersuchungen durchgeführt.

# 14. Finanzierung, ideelle Unterstützung, institutionelle Verbindungen, mögliche Interessenskonflikte, Anreize für Versuchspersonen, Entschädigungen

Die Studie wird durch die SRH Förderstiftung finanziell unterstützt. Die Fördersumme ist im Verhältnis zum Aufwand des Projektes vergleichsweise gering. Darüber hinaus gibt es keine weitere finanzielle Unterstützung von Dritten. Die Stimulationsgeräte vom Typ DCS Mobile wurden von der Herstellerfirma NeuroConn über die Firma Hasomed regulär käuflich erworben. Um eine effektive doppelte Verblindung zu gewährleisten, werden die Randomisierungscodes als Dienstleistung durch die Firma NeuroConn zur Verfügung gestellt. Darüber hinaus besteht keine Kooperation mit NeuroConn. Es liegen keine Interessenskonflikte vor. Die Patienten enthalten keine Aufwandsentschädigung für ihre Teilnahme. Über die Betriebshaftpflicht-Versicherung (Versicherer: R + V Allgem. Versicherung AG) ist die verschuldensabhängige Haftung (= gesetzliche Haftpflicht) der SRH Klinikum Karlsbad-Langensteinbach GmbH bei der Durchführung von klinischen Studien mitversichert. Die Versicherungssummen des SRH-Konzernvertrages betragen je Schadenfall: 7.500.000,- € pauschal für Personen- und Sachschäden und 200.000,- € für Vermögensschäden.

# 15. Literaturverzeichnis

- Aschenbrenner, S., Kaiser, S., Pfüller, U., Roesch-Ely, D. & Weisbrod, M. (2012). *Wiener Testsystem: Testset Kognitive Basistestung (CogBat)*. Mödling: Schuhfried.
- Beblo, T., Kunz, M., Lautenbacher, S., Albert, A. & Aschenbrenner, S. (2011). *Wiener Testsystem: Fragebogen zur geistigen Leistungsfähigkeit*. Mödling: Schuhfried.
- Bikson, M., Grossman, P., Thomas, C., Zannou, A. L., Jiang, J., Adnan, T.et al. (2016). Safety of Transcranial Direct Current Stimulation: Evidence Based Update 2016. *Brain Stimul, 9*(5), 641-661.
- Bodatsch, M. (2014). Transkranielle Gleichstromstimulation bei psychischen Störungen. In J. Kuhn & W. Gaebel (Hrsg.), *Therapeutische Stimulationsverfahren für psychiatrische Erkrankungen* (S. 155-169). Stuttgart: Kohlhammer.
- Brunoni, A. R. & Vanderhasselt, M. A. (2014). Working memory improvement with non-invasive brain stimulation of the dorsolateral prefrontal cortex: a systematic review and meta-analysis. *Brain Cogn, 86*, 1-9.
- Cicerone, K., Levin, H., Malec, J., Stuss, D. & Whyte, J. (2006). Cognitive rehabilitation interventions for executive function: moving from bench to bedside in patients with traumatic brain injury. *J Cogn Neurosci, 18*(7), 1212-1222.
- Cohen, D. (1988). *Statistical Power Analysis for the Behavioral Sciences* (2nd). Hillsdale: Lawrence Erlbaum Associates.
- Dickinson, D., Ramsey, M. E. & Gold, J. M. (2007). Overlooking the obvious: a meta-analytic comparison of digit symbol coding tasks and other cognitive measures in schizophrenia. *Arch Gen Psychiatry, 64*(5), 532-542.
- Dockery, C. A., Hueckel-Weng, R., Birbaumer, N. & Plewnia, C. (2009). Enhancement of planning ability by transcranial direct current stimulation. *J Neurosci, 29*(22), 7271-7277.
- Exner, C. & Lincoln, T. (2012). *Neuropsychologie schizophrener Störungen*. Göttingen: Hogrefe.
- Faul, F., Erdfelder, E., Lang, A. G. & Buchner, A. (2007). G*Power 3: a flexible statistical power analysis program for the social, behavioral, and biomedical sciences. *Behav Res Methods, 39*(2), 175-191.
- Fisher, M., Herman, A., Stephens, D. B. & Vinogradov, S. (2016). Neuroscience-informed computer-assisted cognitive training in schizophrenia. *Ann N Y Acad Sci, 1366*(1), 90-114.
- Fregni, F., Boggio, P. S., Nitsche, M., Bermpohl, F., Antal, A., Feredoes, E.et al. (2005). Anodal transcranial direct current stimulation of prefrontal cortex enhances working memory. *Exp Brain Res, 166*(1), 23-30.
- Glahn, D. C., Laird, A. R., Ellison-Wright, I., Thelen, S. M., Robinson, J. L., Lancaster, J. L.et al. (2008). Meta-analysis of gray matter anomalies in schizophrenia: application of anatomic likelihood estimation and network analysis. *Biol Psychiatry, 64*(9), 774-781.
- Harvey, P. D., Green, M. F., Bowie, C. & Loebel, A. (2006). The dimensions of clinical and cognitive change in schizophrenia: evidence for independence of improvements. *Psychopharmacology (Berl), 187*(3), 356-363.
- Hautzinger, M., Keller, F. & Kühner, C. (2006). *Beck Depressions- Inventar (BDI-II). Revision*. Frankfurt/Main: Harcourt Test Services.
- Hill, A. T., Fitzgerald, P. B. & Hoy, K. E. (2016). Effects of Anodal Transcranial Direct Current Stimulation on Working Memory: A Systematic Review and Meta-Analysis of Findings From Healthy and Neuropsychiatric Populations. *Brain Stimul, 9*(2), 197-208.
- Hoy, K. E., Arnold, S. L., Emonson, M. R., Daskalakis, Z. J. & Fitzgerald, P. B. (2014). An investigation into the effects of tDCS dose on cognitive performance over time in patients with schizophrenia. *Schizophr Res, 155*(1-3), 96-100.
- Kanning, U. P. (2009). *Inventar Sozialer Kompetenzen (ISK)*. Göttingen: Hogrefe.
- Kuhl, J. & Kazén, M. (2009). *Persönlichkeits-Stil-und-Störungs-Inventar (PSSI). Manual.* (Bd. 2nd Ed.). Göttingen: Hogrefe.
- Lehrl, S. (2005). *Mehrfachwahl-Wortschatz-Intelligenztest MWT-B* (Bd. 5. Auflage). Balingen: Spitta Verlag.
- Leucht, S., Vauth, R., Olbrich, H. M. & Jäger, M. (2014). Schizophrenien und andere psychotische Störungen. In M. Berger (Hrsg.), *Psychische Erkrankungen : Klinik und Therapie* (5. ed., S. 301 - 358). München: Elsevier.
- Mervis, J. E., Capizzi, R. J., Boroda, E. & MacDonald, A. W., 3rd. (2017). Transcranial Direct Current Stimulation over the Dorsolateral Prefrontal Cortex in Schizophrenia: A Quantitative Review of Cognitive Outcomes. *Front Hum Neurosci, 11*, 44.
- Mesholam-Gately, R. I., Giuliano, A. J., Goff, K. P., Faraone, S. V. & Seidman, L. J. (2009). Neurocognition in first-episode schizophrenia: a meta-analytic review. *Neuropsychology, 23*(3), 315-336.
- Minzenberg, M. J., Laird, A. R., Thelen, S., Carter, C. S. & Glahn, D. C. (2009). Meta-analysis of 41 functional neuroimaging studies of executive function in schizophrenia. *Arch Gen Psychiatry, 66*(8), 811-822.
- Miyake, A., Friedman, N. P., Emerson, M. J., Witzki, A. H., Howerter, A. & Wager, T. D. (2000). The unity and diversity of executive functions and their contributions to complex "Frontal Lobe" tasks: a latent variable analysis. *Cogn Psychol, 41*(1), 49-100.
- Montgomery, S. A. & Asberg, M. (1979). A new depression scale designed to be sensitive to change. *Br J Psychiatry, 134*, 382-389.
- Müller, S. V. (2013). *Störungen der Exekutivfunktionen*. Göttingen: Hogrefe.
- Müller, S. V., Hildebrandt, H. & Münte, F. (2004). *Kognitive Therapie bei Störungen der Exekutivfunktionen - ein Therapiemanual*. Göttingen: Hogrefe.
- Nienow, T. M., MacDonald, A. W., 3rd & Lim, K. O. (2016). TDCS produces incremental gain when combined with working memory training in patients with schizophrenia: A proof of concept pilot study. *Schizophr Res, 172*(1-3), 218-219.
- Nitsche, M. A. & Paulus, W. (2000). Excitability changes induced in the human motor cortex by weak transcranial direct current stimulation. *J Physiol, 527 Pt 3*, 633-639.
- Nitsche, M. A. & Paulus, W. (2001). Sustained excitability elevations induced by transcranial DC motor cortex stimulation in humans. *Neurology, 57*(10), 1899-1901.
- Nuechterlein, K. H., Subotnik, K. L., Green, M. F., Ventura, J., Asarnow, R. F., Gitlin, M. J.et al. (2011). Neurocognitive predictors of work outcome in recent-onset schizophrenia. *Schizophr Bull, 37 Suppl 2*, S33-40.
- Pope, P. A., Brenton, J. W. & Miall, R. C. (2015). Task-Specific Facilitation of Cognition by Anodal Transcranial Direct Current Stimulation of the Prefrontal Cortex. *Cereb Cortex, 25*(11), 4551-4558.
- Rindermann, H. (2009). *Emotionaler Kompetenz Fragebogen (EKFS)*. Göttingen: Hogrefe.
- Smith, R. C., Boules, S., Mattiuz, S., Youssef, M., Tobe, R. H., Sershen, H.et al. (2015). Effects of transcranial direct current stimulation (tDCS) on cognition, symptoms, and smoking in schizophrenia: A randomized controlled study. *Schizophr Res, 168*(1-2), 260-266.
- Tsang, H. W., Leung, A. Y., Chung, R. C., Bell, M. & Cheung, W. M. (2010). Review on vocational predictors: a systematic review of predictors of vocational outcomes among individuals with schizophrenia: an update since 1998. *Aust N Z J Psychiatry, 44*(6), 495-504.
- Wittchen, H.-U., Zaudig, M. & Fydrich, T. (1997). *Strukturiertes Klinisches Interview für DSM-IV.* Göttingen Hogrefe.
